# Design of ion channel blocking, toxin-like Kunitz inhibitor peptides from the tapeworm, *Echinococcus granulosus*, with potential anti-cancer activity

**DOI:** 10.1038/s41598-023-38159-w

**Published:** 2023-07-15

**Authors:** Zahra Rashno, Elham Rismani, Jahan B. Ghasemi, Mehdi Mansouri, Mohammad Shabani, Ali Afgar, Shahriar Dabiri, Farahnaz Rezaei Makhouri, Abbas Hatami, Majid Fasihi Harandi

**Affiliations:** 1 Research Center for Hydatid Disease in Iran, Afzalipour School of Medicine, Kerman University of Medical Sciences, Kerman, 7616914115 Iran; 2 Molecular Medicine Department, Biotechnology Research Center, Pasteur Institute of Iran, Tehran, Iran; 3 Faculty of Chemistry, School of Sciences, University of Tehran, Tehran, Iran; 4 Department of Agricultural Biotechnology, Faculty of Agriculture, Shahid Bahonar University of Kerman, Kerman, Iran; 5 Neuroscience Research Center, Institute of Neuropharmacology, Kerman University of Medical Sciences, Kerman, Iran; 6 Pathology and Stem Cell Research Center, Afzalipour School of Medicine, Kerman University of Medical Sciences, Kerman, Iran

**Keywords:** Drug development, Liver cancer, Parasite physiology

## Abstract

Over-expression of K+ channels has been reported in human cancers and is associated with the poor prognosis of several malignancies. EAG1, a particular potassium ion channel, is widely expressed in the brain but poorly expressed in other normal tissues. Kunitz proteins are dominant in metazoan including the dog tapeworm, *Echinococcus granulosus*. Using computational analyses on one A-type potassium channel, EAG1, and in vitro cellular methods, including major cancer cell biomarkers expression, immunocytochemistry and whole-cell patch clamp, we demonstrated the anti-tumor activity of three synthetic small peptides derived from *E. granulosus* Kunitz4 protease inhibitors. Experiments showed induced significant apoptosis and inhibition of proliferation in both cancer cell lines via disruption in cell-cycle transition from the G0/G1 to S phase. Western blotting showed that the levels of cell cycle-related proteins including P27 and P53 were altered upon kunitz4-a and kunitz4-c treatment. Patch clamp analysis demonstrated a significant increase in spontaneous firing frequency in Purkinje neurons, and exposure to kunitz4-c was associated with an increase in the number of rebound action potentials after hyperpolarized current. This noteworthy component in nature could act as an ion channel blocker and is a potential candidate for cancer chemotherapy based on potassium channel blockage.

## Introduction

Cancer is one of the leading causes of death in most countries across the globe. The new cancer cases worldwide have been estimated at 19.3 million in 2020 with about 10 million cancer-related deaths^1^. Cancer is characterized by continuous cell proliferation, angiogenesis, immortality and resistance to cell death, durability to growth suppressors, and enhanced metastatic properties. Cancer cells exploit various signaling mechanisms and functionally different proteins. An important role for ion channels has recently been recognized in all pathophysiological phenotypes of malignant growth, initiation, and progression. It is well-known that a transient hyperpolarization occurs at the exit of the G1 phase in several cancer cell types, and significant depolarization has been documented in tumor cells compared to non-tumor cells^2^. Current evidence indicates that K+ channels significantly contribute to the control of apoptosis and proliferation of different cancer cells^3^. Pharmacological blockade of voltage-gated potassium channels (Kv) has also resulted in inhibited cell proliferation^4^. Overexpression of different Kv channels has been shown to mark human blood, breast, colon, and prostate cancers, among others^5^.

Recent studies have suggested that several K + channel subtypes are differently expressed, and they contribute to the regulation of the biological behavior of cells, e.g. the aggressiveness of leiomyosarcoma^6,7^, epilepsy progression^8^, and the invasion and migration of endometrial carcinoma^9^. Therefore, some malignant tumors have been categorized as “K + Channel Diseases,” designating K+ channels as promising therapeutic targets^10,11^. Accumulating evidence has also revealed that various K+ channels, including Kv, KCa2+, K2P, and Kir channels, are abnormally expressed in cancer cells^12,13^.

It is well-documented that many tumor cells and cancers show high Kv10.1 expression levels and is critical for malignant cell progress^15^. EAG1 gene encodes the Kv10.1, a member of the EAG family of voltage-gated potassium channels and is believed to play a role in tumor cell proliferation. High levels of Kv10.1 ion channel expression have been demonstrated in the neural system; however, it has been shown that it is also expressed in human non-neurological cancers^12^. The progression of tumor cells expressing Kv10.1 is enhanced by molecular mechanisms involving different cellular pathways, which indicates that protein–protein interactions are essential in the role of Kv10.1 in cell proliferation and oncogenesis^14^. Several studies on oncogenesis have provided interesting findings on the Kv10.1 role in regulating EAG1 expression at transcriptional and post-transcriptional levels, presenting it as an attractive target for cancer therapy.

The multigene family of Kunitz-type inhibitors are present in all metazoan organisms as a class of serine protease inhibitors. Moreover, several Kunitz proteases, known as Kunitz-type toxins (KTTs), act as ion channel blockers^15^. They have been identified as main components of the poisonous animal venoms including spiders, ticks, snakes, sea anemones, scorpions, Hainan cascade-frogs and conid snails^16–19^. KTTs specifically impair various physiological processes, including host defense, fibrinolysis, blood coagulation, and action potential transduction, by blocking voltage-gated potassium channels^15^. Potassium channel blocking induced by dendrotoxins (DTXs) of the green mamba snake enhances acetylcholine release at neuromuscular junctions with no protease inhibitory activity^20^.

A couple of studies on *Schistosoma mansoni* Kunitz type protease inhibitor (SmKI-1) have demonstrated different protease inhibitory activities. Morais et al. showed that SmKI-1 inhibited neutrophil elastase and trypsin and had an anti-inflammatory effect on various inflammatory disorder models by inhibiting neutrophil migration and function^21^. In another model, SmKI-1 inhibited plasma kallikrein and caused delayed blood clot formation by increasing activated partial thromboplastin/prothrombin time^22^.

Eight Kunitz proteins have been demonstrated in the dog tapeworm, *Echinococcus granulosus*. Kunitz inhibitors, mostly expressed in pepsin-treated protoscoleces, play a significant role in *E. granulosus*–dog crosstalk, particularly in the initial phases of dog infection^23^. Interestingly, two types of *E. granulosus* Kunitz inhibitors, i.e. Kunitz1 and Kunitz4, did not inhibit peptidases, and structural features related to the activities in Kunitz cation-channel blocking have also been demonstrated in the transcriptomes from *E. multilocularis* and *Taenia solium*, the two major tapeworms of medical importance^24^. This suggests that families of single domain Kunitz inhibitors are present in closely related platyhelminths. Ranasinghe et al.^25^ showed that recombinant EgKI-1 (*Echinococcus granulosus* Kunitz inhibitor 1) inhibits the in vitro cancer cell growth and migration in human breast, melanoma, and cervical cancer cell lines in a dose-dependent manner without affecting normal cell growth.

**Figure 1:**
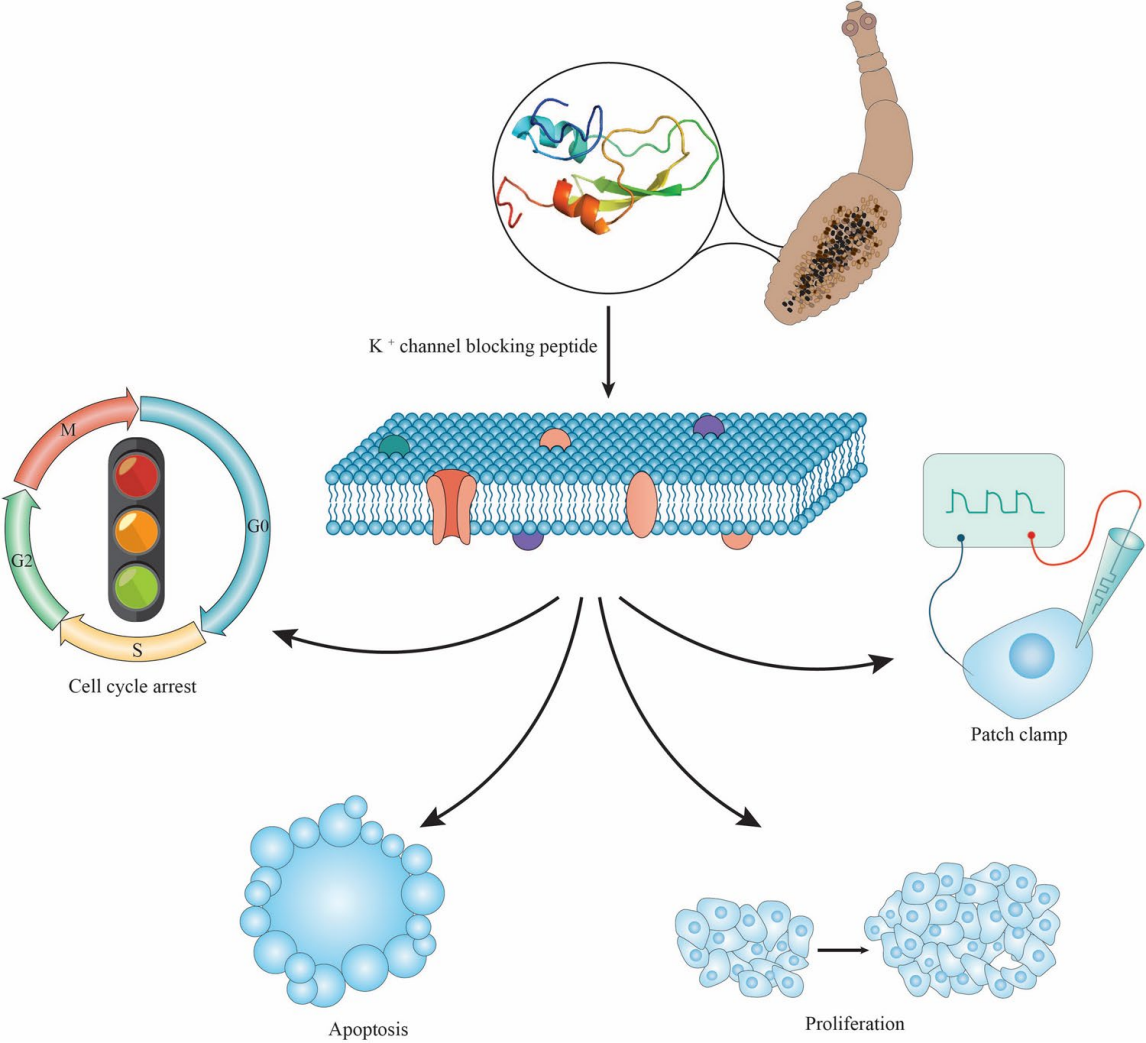
Schematic representation of inhibitory investigation of Kunitz4 derived from *Echinococcus granulosus* on different biological features of tumor cells including proliferation, apoptosis, cell cycle, and cancer-related voltage-gated K^+^ -channels.

In the present study, we characterized the anti-cancer activity of three synthetic small peptides derived from *E. granulosus* Kunitz4 protease inhibitor using computational modeling as well as in vitro cellular methods and biological assays (Fig. 1). In addition, we investigated protein–protein interactions (PPI) and *E. granulosus* Kunitz4 affinity behavior for the EAG1 (Kv10.1) channel.

## Results

### Sequence analysis of EgKU4 protein and model validation

The current study predicted the 3D structure of Kunitz4 using the full-length mature sequence of *E. granulosus* Kunitz4, the constituent of 84 amino acids, and the sequence of 1dtx, 1dem, 1dtk, and1tfx (Figure S1). Modeller 9.22 was employed to compute structural models using the BLOSUM62 similarity matrix. The top 10 from among 10,000 constructed models, based on the lowest discrete optimized potential energy (DOPE) score, were selected for validation of stereochemical features. Valuation of model reliability was done in terms of Z-scores. Analysis of Ramachandran plot of the 3D structures from RAMPAGE showed that 94.7%, 3%, 2%, and 0% of residues were placed in the most favored, additionally allowed, generously allowed, and disallowed regions, respectively (Fig. S2, Supplementary Table 1). The z-score for the predicted model was − 4.4 with the ProSA web server, indicating an acceptable model quality compared with experimentally validated protein structures (Figure S2e). Additionally, the results of a secondary structure alignment demonstrated a significant secondary structure compatibility between Kunitz4 and the template model (Figure S2c). To assess the environment of each residue in the model, the compatibility of 3D-1D structures was evaluated using Verify 3D score.

### Comparable interaction of Kunitz4 and EAG1 in three distinct sites

**Figure 2:**
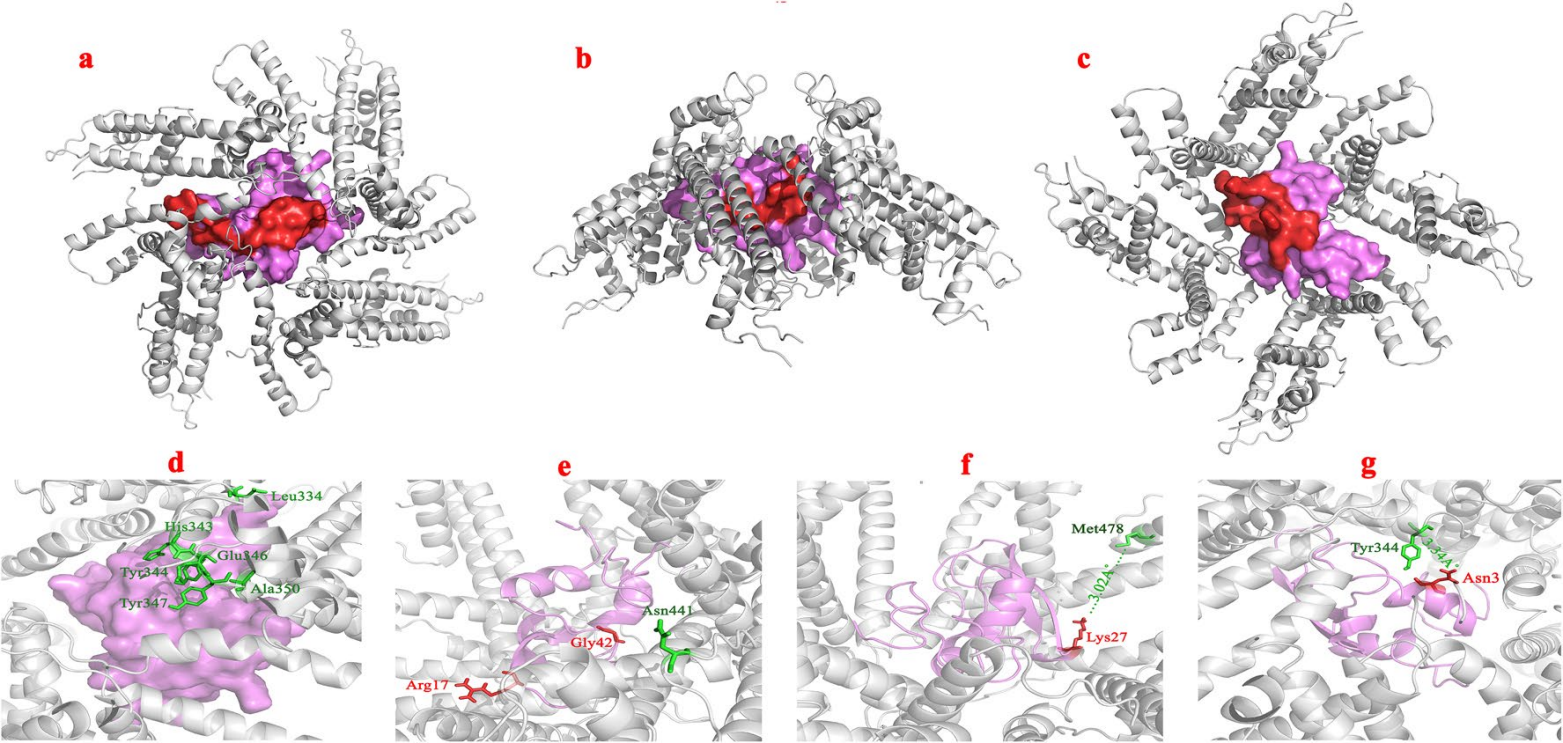
Structural analysis of binding sites of the Kunitz4-EAG1 complex acquired from docking studies: (**a**) the docking poses three main distinct binding sites (red) interacting with Kunitz4 on the N-terminal and C-terminal of EAG1 protein, comprised of ranges of 18–35, 46–55 (**b**), and 65–84 (**c**) amino acids. Kunitz4 and EAG1 are colored in magenta and grey, respectively. (**d**) Graphical depiction of the interaction of the putative binding site of EAG1, located close to the selectivity filter between S5 and S6 segments. (**e**) Close-up view of Asn441 in chain A of EAG1 interacting with Arg37 and Gly62 of Kunitz4. (**f**) The critical Lys47 of Kunitz4 involved in EAG1 Met478 (chain D) with the bond length of 3.02 Å in hydrogen bond. (**g**) Asn23 formed a hydrogen bond to Tyr344 (D chain) of EAG1 with a length of 3.34 Å. Residues of EAG1 and Kunitz4 involved in the interaction are colored in green and red, respectively.

Findings of the study indicated that the putative binding site of EAG1, located close to the selectivity filter between S5 and S6 segments and consisting of Tyr423, Met478, Tyr347, Lys340, Glu346, Ala350, Thr430, His364, Tyr344, and His343, belongs to the extracellular opening of the pore (Fig. 2d). The docking poses revealed three distinct binding sites interacting with Kunitz4 on the N-terminal and C-terminal of the EAG1 protein comprised of ranges of 18–35, 46–55, and 65–84. These sites were probably involved in the channel interaction, so these amino acids were selected for the design of peptides (Fig. 2a–c). Regarding the binding mode, analysis of the docking revealed that the Kunitz4 binding was centered, the number of hydrogen bonds was considerable, and the hydrophobic interactions were more dominant than other interactions. Almost all of the interaction patterns showed that they shared several common conserved residues based on multiple sequence alignment in their interactions. Based on molecular docking, consistency was found between the predicted active and passive interfacing amino acids and the functional amino acids in either EAG1 or Kunitz4. The lowest energy of binding for the EAG1-Kunitz4 complex was − 3149.6 and − 659.452 kcal/mol, for Cluspro and HADDOCK, respectively (Table 1). We found the critical Lys47 of Kunitz4 involved in Met478 (chain D) with the bond length of 3.02 Å in a hydrogen bond in the S5–S6 domain. As shown in Fig. 2, binding mode in Fig. 2e places Kunitz4 Arg37 and Gly62 side chains in close proximity to Asn441 (A chain) of EAG1; with a fully elongated Kunitz4, Asn23 formed a hydrogen bond to Tyr344 (D chain) of EAG1 with a length of 3.34  Å. Dramatically, the peptides revealed a very low binding free energy in the complex with EAG1 (Table 1). Peptide-EAG1 docking turned out to be compatible with the mode and affinity of the Kunitz4 and EAG1 interaction (Figure S3).

**Table 1:** Molecular docking results of KCNH1-Kunitz4 and Kunitz4 peptides.

	HADDOCK z-score	Number of H-bonds	Number of hydrophobic bonds	ΔG (kcal/mol)	Kd (M) at 37.0 ℃	Lowest energy weighted score	ΔG (kcal/mol)	Kd (M) at 37.0 ℃
	HADDOCK					Cluspro		
Kunitz4-KCNH1	− 1	14	31	− 8.8	6.60E−07	− 3149.6	− 18.1	1.70E−13
Kunitz4-a-KCNH1	− 1	2	9	− 6.9	3 × 10^–6^	− 815.7	− 8.1	1.5 × 10^–6^
Kunitz4-b-KCNH1	− 1	5	–	− 7.1	1.75 × 10^–6^	− 1673.8	− 6.8	6 × 10^–6^
Kunitz4-c-KCNH1	− 1	7	2	− 9.2	2.5 × 10^–6^	− 1372.4	− 7.4	2 × 10^–6^

### Stability of predicted model and Kunitz4-EAG1 complex after MD simulation

**Figure 3:**
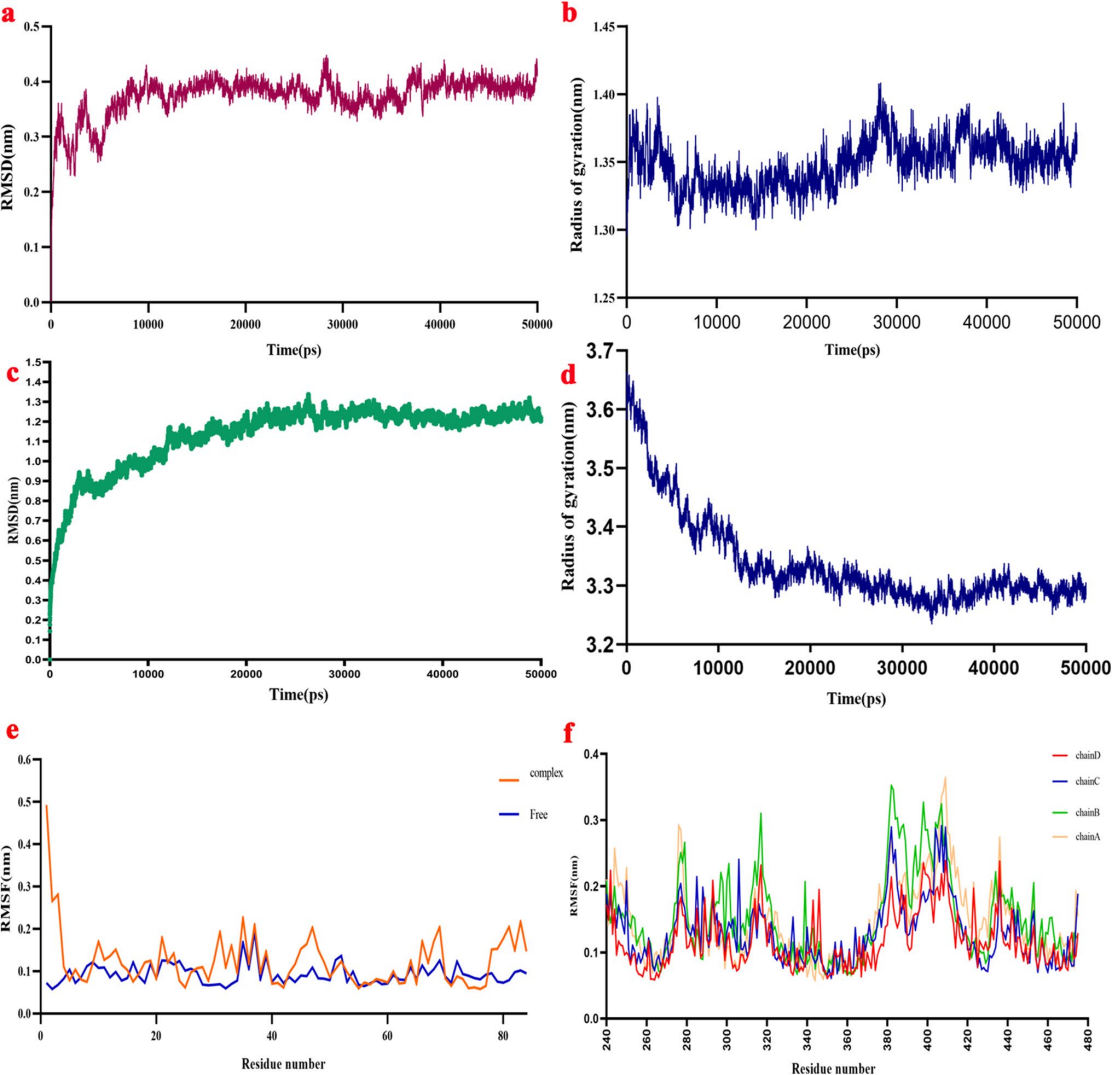
Molecular dynamics (MD) simulation analysis of the Kunitz4 model and the Kunitz4-EAG1 complex in the period of 50,000 ps. (**a**) RMSD plot of backbone Cα atoms of Kunitz4 that showed the steady state of the model from 28,000 ps. (**b**) The radius of gyration (Rg) plot that represented stability and compactness of Kunitz4 from 26,000 ps of simulation. (**c**) RMSD plot of Kunitz4-EAG1 complex in 50,000 ps of MD simulation. (**d**) Rg plot of the Kunitz4-EAG1 showed compact and relaxed states of the complex around 15,000 ps of simulation. (**e**) RMSF plot of Kunitz4 in the complex with EAG1 and in the free form. (f) RMSF plot of EAG1 showing fluctuations around 0.1 nm in all chains.

The structural stability of the model was studied using molecular dynamics (MD) simulation. The output was analyzed to determine conformational changes against the initial structure by root mean square deviation (RMSD) based on the structure of the backbone. It is used in protein structure predictions to assess the match between the modeled and X-ray/NMR protein structure and often computed using only the Cα or backbone heavy atoms of each amino acid. The RMSD plot of the Kunitz4 structure fluctuated around 28,000 ps of the simulation and then reached a steady state within 0.3–0.4 nm of RMSD up to 50,000 ps (Fig. 3a). Radius of gyration (Rg) was calculated to estimate the compression and folding of the protein structure during the simulation (Fig. 3b). The Rg value of Kunitz4 increased slightly from 1.30 nm at the beginning to 1.36 nm around the 26,000th ps of simulation, and stability was maintained until the end of the simulation. This demonstrated that the Kunitz4 structure reached a stable and compact conformation during the simulation. To recognize the dynamics and function relationships of protein movements, the residue-based root mean square fluctuation (RMSF) values of the backbone Cα were compared. A large RMSF value indicated a flexible region with motion, while a low RMSF value proposed a rigid region and minimal motion. During simulation, the RMSF of Kunitz4 was less than 0.2 nm for all residues, indicating low changes in the protein structure.

**Figure 4:**
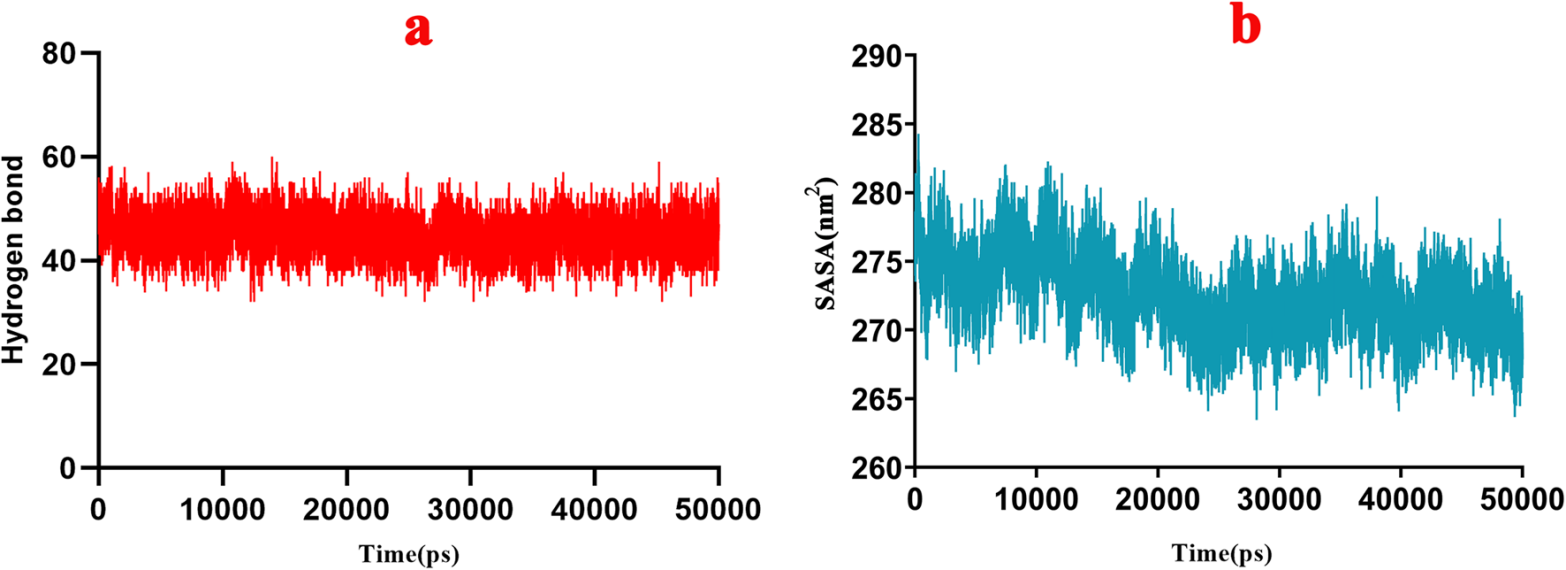
Hydrogen bond and SASA analysis of Kunitz4-EAG1 complex during MD simulation. (**a**) The total number of hydrogen bonds were variable between 40 and 60 peptides for Kunitz4-EAG1 complex in binding during of MD simulation. (**b**) SASA plot of Kunitz4-EAG1 complex analysis showed that accessible area in the start of simulation was around 285 nm^2^, whereas at the end of simulation the area was finally lowered to the range of 265 nm^2^.

Molecular dynamics simulations for Kunitz4-EAG1 indicated the stability of the formed intermolecular interactions during the MD simulation, RMSD showing the stability of the complex during simulation with fluctuation of 0.3–0.8 nm (Fig. 3c). The Rg plot showed that Kunitz4-EAG1 became compact and relaxed at 30,000th ps of the simulation (Fig. 3d). The RMSF plot of EAG1 revealed slight fluctuations during the simulation. This could be interpreted as the higher stability of the four chains compared to Kunitz4, indicating approximately the same fluctuations in both EAG1 binding sites (Fig. 3f). In contrast, the RMSF plot of Kunitz4 in complex with EAG1 showed that the selected region for peptides fluctuated slightly in complex in comparison with free Kunitz4, at least in the two binding sites of EAG1 and Kunitz4, while binding sites between 64 and 75 exhibited minimal fluctuations (Fig. 3e). The number of hydrogen bonds is an index of the quality of the interaction. The total number of hydrogen bonds in the binding site during simulation varied between 40 and 60, and these findings validated the previous docking results (Fig. 4a). Accessible surface area solvent (SASA) was calculated for the interface of EAG1 and Kunitz4. Higher SASA values indicated more exposed amino acids in solvent, whereas lower values indicated more concealed residues in interaction. SASA plot analysis determined that the accessible area at the start of simulation was around 285 nm^2^, while the area was ultimately lowered to the range of 260–265 nm^2^ at the end of simulation (Fig. 4b).

### Molecular mechanics/Poisson–Boltzmann surface area (MM-PBSA) calculation and binding energies

**Figure 5:**
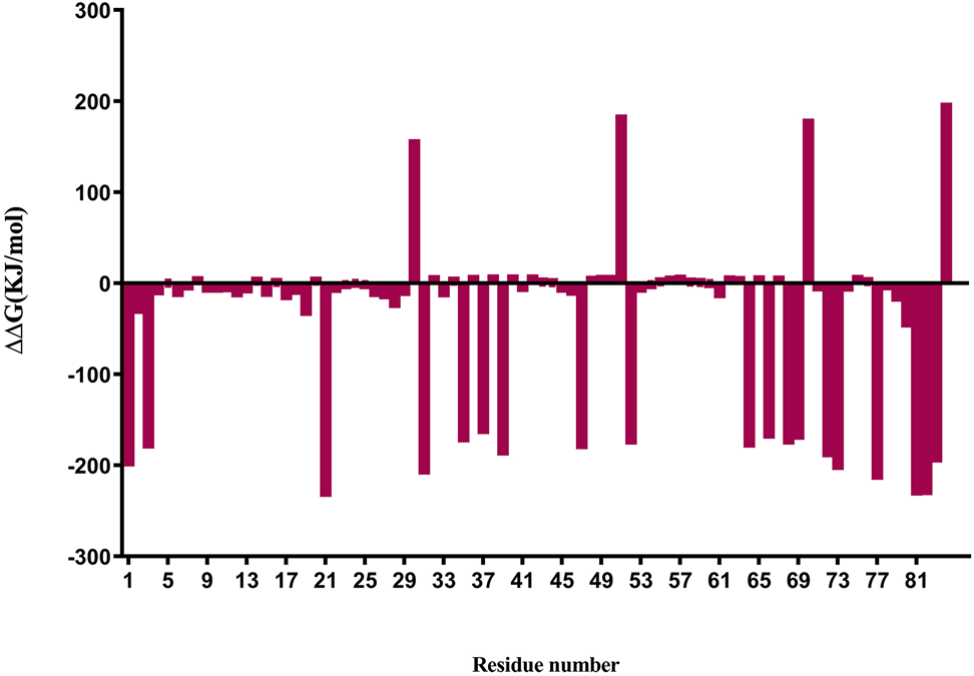
Calculated and experimental effects on the blocking Kunitz4 affinity for alanine mutations of EAG1. The calculated results are normalized values of ΔΔGbinding. ΔΔG is defined as ΔGmut−ΔGwt, where ΔGwt and ΔGmut are the binding free energies upon complex formation of the wild-type and alanine-mutated proteins, respectively.

The EAG1-Kunitz4 complex was analyzed by considering residues contributed favorably to ΔE_binding_ by means of energy decomposition calculation. Hot spots were identified by looking at residues that contributed significantly to complex stabilization. The area that showed the highest stabilization was identified in three specific regions located in the contact surface between EAG1 and Kunitz4. Supplementary Dataset file 2 shows residues of Kunitz4 that contributed to the stabilization of the complex. As shown in Fig. 5, in fragment kunitz4-a, the residues Arg21, Val22, Asn26, Leu27, Pro28, Ile29, Lys31, Gln33, and Arg35, in fragment kunitz4-b the residues Pro45, Ser46, Lys47, Arg52, and Phe53, and in fragment kunitz4-c, the residues Arg64, Lys66, Lys68, Arg69, Lys72, and Arg73, had more than 10 kj/mol of free energy contributed to their binding to the protein.

### Binding affinities of the designed peptides by molecular docking

Peptides designed from Kunitz4 were evaluated based on their binding mode to EAG1 by ClusPro and HADDOCK web servers. Models with interactions were selected based on the lowest binding energy, higher hydrogen bond, and higher binding affinity to EAG1. Complexes formed from kunitz4-a and kunitz4-c were more stable with lower binding energy values of ΔG = − 8.1 kcal/mol and ΔG = − 7.4 kcal/mol, respectively, compared to the kunitz4-b complex with ΔG = − 6.8 kcal/mol. Likewise, measuring dissociation constant (Kd) showed that peptides a and c had lower kd compared to peptide a, with 1.5 × 10^–6^ M and 2 × 10^–6^ M vis-a-vis 6 × 10^–6^ M, respectively (Table 1). These derived peptides also showed higher numbers of hydrogen bonds and lower binding energy compared with peptide b.

### Peptides inhibit HepG2 and HT29 cell proliferation

**Figure 6:**
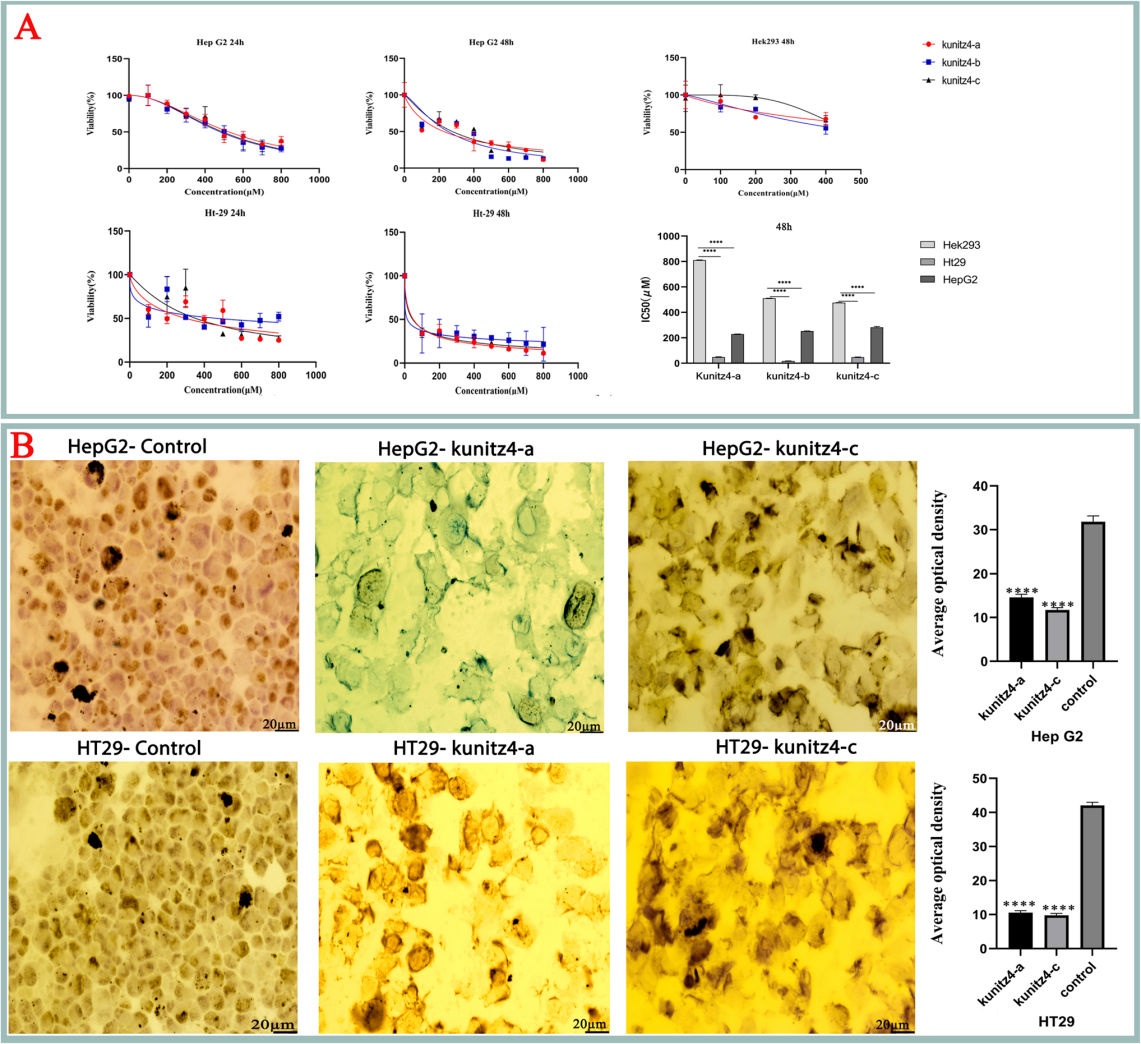
(**A**) The Effects of various concentrations of kunitz4-a, kunitz4-b, and kunitz4-c on the viability of two tumor cell lines, hepatocellular carcinoma (HepG2) and colorectal adenocarcinoma cells (HT29) as well as the normal cell line (Hek293). The error bars represent mean ± SEM of the triplicate measurements. (**B**) Ki-67 expression analysis in HT29 and HepG2 cell lines treated with kunitz4-a and kunitz4-c for 24 h as compared to the untreated controls. Scale bar = 20 µM. Results were obtained as mean ± SEM densitometry index of three independent experiments performed in triplicate. ****p < 0.0001.

MTT assays were used to test the effects of the three peptides on the proliferation of cancerous HT29 and HepG2 cell lines as well as the normal Hek293 cell line (Fig. 6, Figure S4). A significant inhibition in the tumor cell activity of kunitz4-a, kunitz4-b, and kunitz4-c was documented in various concentrations (from 0 μM up to 800 μM). The half maximal inhibitory concentrations (IC50) for the HepG2 cell line treated for 24 h with kunitz4-a, kunitz4-b, and kunitz4-c were 525.7 µM, 480.7 µM, and 493.1 µM, respectively. These figures for the HT29 cell line were 309.1 µM, 446.6 µM, and 354.1 µM, respectively. The IC50 of kunitz4-b on HT29 cancer cells was at 15.69 μM concentration, showing a significantly stronger effect than that of the HepG2 cells (*p* =  < 0.0001), (Fig. 6). After 48 h, no significant suppression of normal Hek293 cells displayed in treatment with the peptide kunitz4-c up to 300 μM, however at 400 μM, between 25 and 35% decline in viability were obtained using all the peptides. The optimal amount of IC50 in cancer cells was obtained in the range equal to 251 µM for Hep G2 and 15.69 µM for HT29 in 48 h. Therefore, to investigate cytotoxicity effects of a 48-h study, comparing data of cancerous and normal cell lines was only considered at concentrations in the range of 0–400 μM (Fig. 6).

### Immunocytochemistry staining

The expression of Ki-67 was analyzed in HT29, and HepG2 cell lines for kunitz4-a and kunitz4-c in 100 and 250 µM, respectively. The findings indicated a significant difference in the cells treated with small peptides compared to the untreated controls. As shown by densitometry analysis, the significantly reduced expression of Ki-67 was observed after treatment with the small peptides kunitz4-a and kunitz4-c compared to the untreated cells (*p* < 0.0001, Fig. 6-Panel B).

### Kunitz4 peptides promote apoptotic cell death

**Figure 7:**
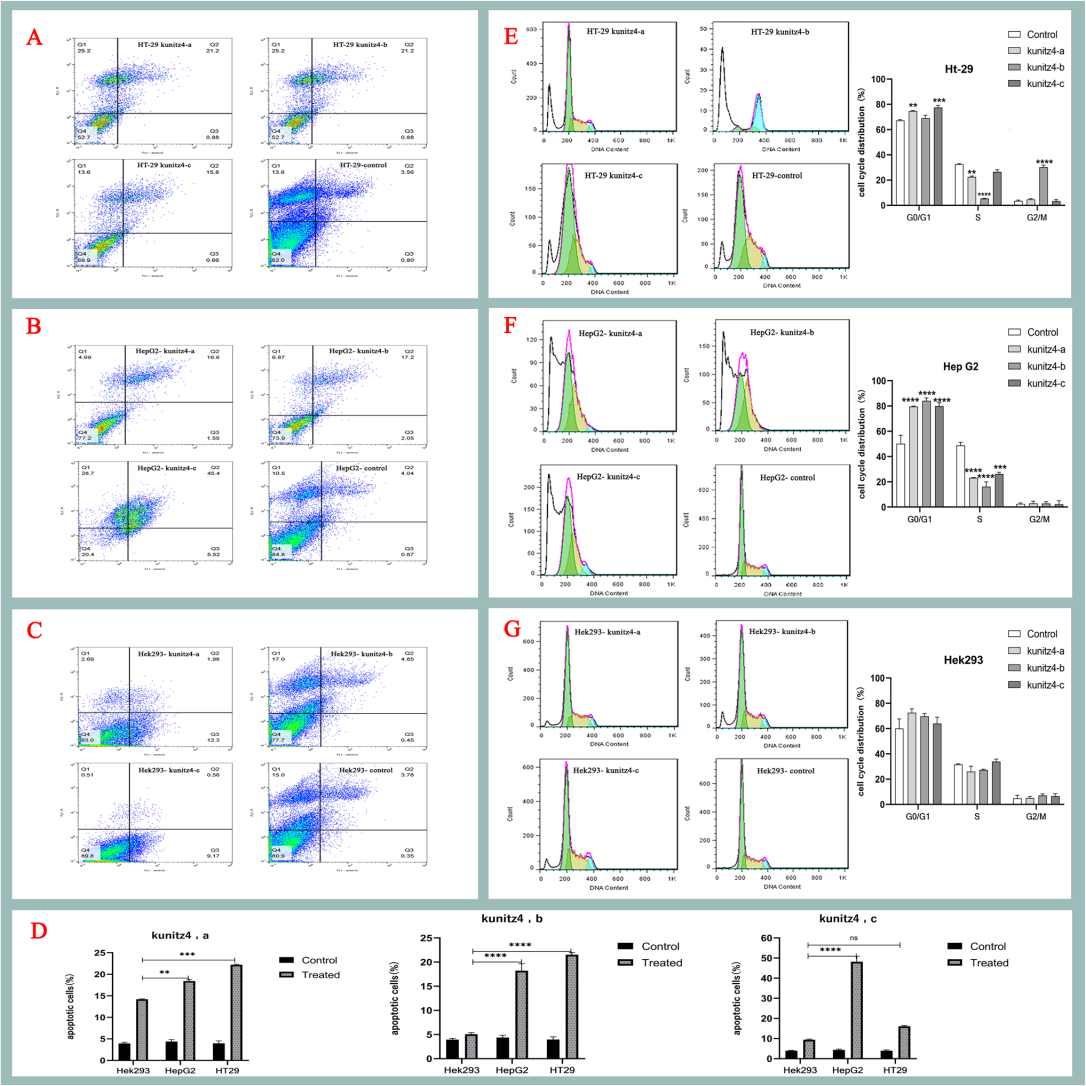
Effects of the three synthetic peptides on externalization of phosphatidyl serine and cell cycle progression in human embryonic kidney cells (Hek293) and the two tumor cell lines, colorectal adenocarcinoma (HT29) and hepatocellular carcinoma (HepG2). (**A**–**C**) The distribution of apoptotic cells stained with annexin V-FITC/PI in dual parametric dot plots of PI fluorescence (Y-axis) versus annexin V-FITC fluorescence (X-axis). (**D**) Apoptotic cell death (%) of the cancerous cell lines compared to the normal Hek293 cells according to each of the three peptidesCell cycle experiments were performed using FACScalibur flow cytometer. Effect of the three peptides on HT29 (**E**), HepG2 (**F**) and Hek293 (**G**) with cell cycle distribution phases. Values are presented as mean ± SEM from at least three independent experiments. The asterisks indicate significant different values compared to the untreated controls (****p ≤ 0.0001,  ***p = 0.0001,  **p = 0.0051).

To examine the apoptotic effects, HT29 and HepG2 cell lines were treated with Kunitz4 peptides and stained with annexin V and PI. As shown in Fig. 7, after 24 h of exposure to 100 μM of each peptide for HT29, and 250 μM for HepG2 and the normal Hek293 cells, a significant increase in apoptosis was observed in HT29 (*p* = 0.005) and HepG2 (*p* = 0.001) cell lines. The ability of kunitz4-c to promote apoptosis was significantly stronger on HepG2 (*p* = 0.0009) than on HT29 cells (*p* = 0.005). The peptides induced low apoptotic effects on the normal cell line compared to the cancerous cells, i.e. we reported 5.3% and 9.73% apoptosis for kunitz-b and kunitz-c, respectively, which are significantly lower than the corresponding values for HepG2 (19.25% and 50.92%) and Ht29 (22.08% and 16.46%). The results for kunitz-b and kunitz-c were more satisfactory than kunitz-a. The maximum amount of apoptosis level was observed in HepG2 occurred under kunitz4-c.

### Derived peptides caused remarkable cell cycle arrest at the G0/G1 phase

To investigate cell cycle arrest involvement in the cell growth blockade, cell cycle alterations in HT29, HepG2, and Hek293 cells following Kunitz4 peptide treatments were evaluated. As shown in Fig. 7, all peptides caused a significant increase in both HT29 and HepG2 cell populations at the G0/G1 phase and S phase along with a decrease in cell population in G2/M compared to the control group (*p* =  < 0.0001), while no remarkable cell cycle effects were found on normal Hek293 cells treated with peptides (*p* = 0.7217). These findings indicated that the small peptides inhibited the growth of HT29 and HepG2 cells with cell cycle arrest at the G0/G1 phase.

### *P53*,* P27* genes are highly expressed in cancer cells treated with kunitz4-a and kunitz4-c

**Figure 8:**
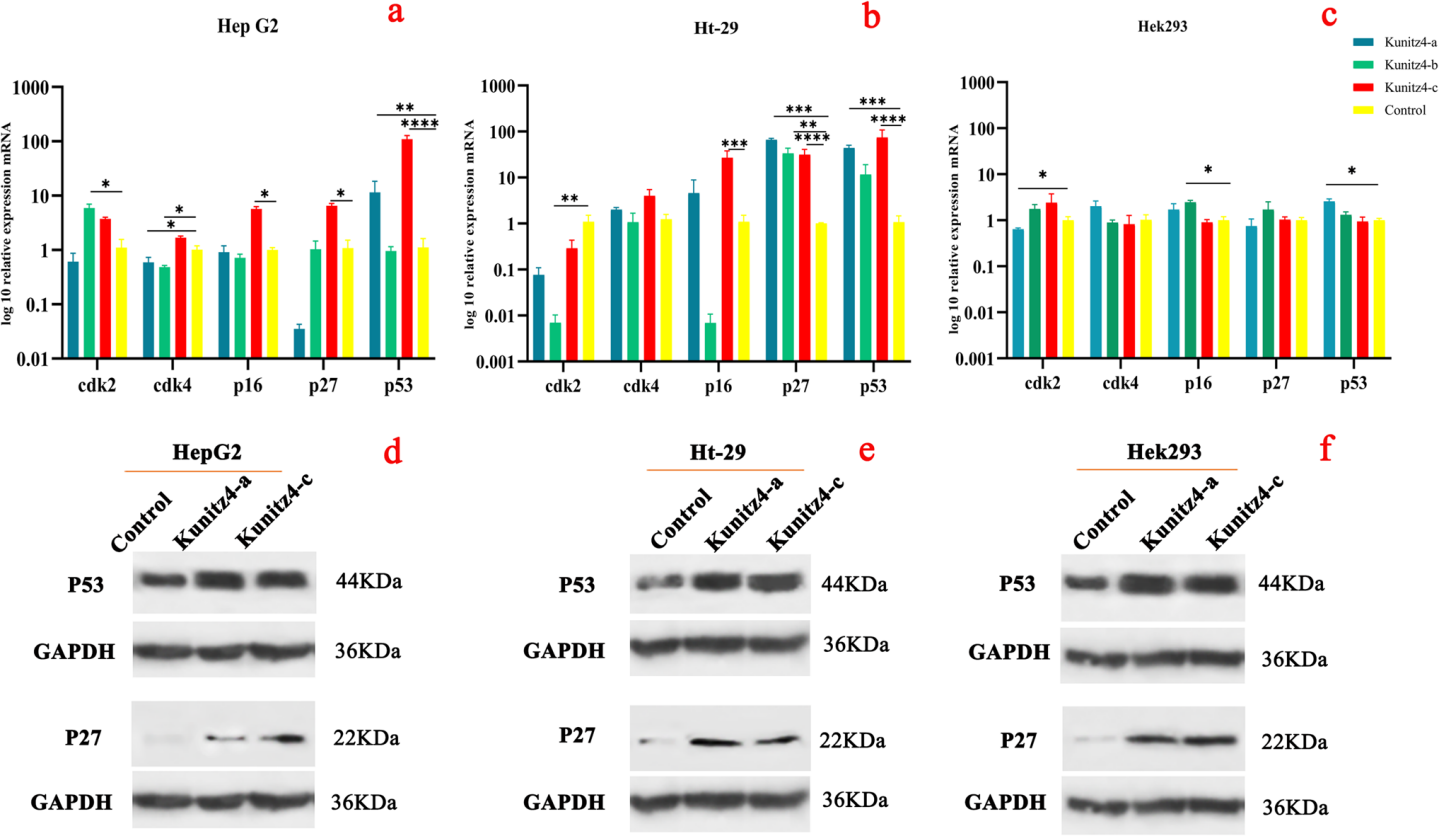
Effects of small peptides on the expression of representative genes and protein markers after 24 h. (**a**–**c**) Relative expression of *CDK2*, *CDK4*, *P16, P27,* and *P53* after 24 h incubation with the small peptides (**a**) HepG2, (**b**) HT29, and (**c**) Hek293. *GAPDH* and *TFG* were used as reference genes for HepG2, and *PGK1* and *GAPDH* were used for the HT29 cell line. (**d**) Western blotting analysis of the effects of the small peptides, kunitz4-a and kunitz4-c, on the protein expression of *P53* and *P27* on Hek293, HepG2, and HT29. *GAPDH* was used as an internal control for optimization. Original blots are presented in Supplementary Fig. 1. The data are the mean value ± SEM. *p < 0.05, **p < 0.01, ***p < 0.001 and ****p < 0.0001.

Expression levels of the five gene markers, i.e. *P53, P27, P16, CDK4,* and *CDK2,* as determined by qRT-PCR are shown in Fig. 8. The relative *P53* mRNA in kunitz4-a was increased by 11 and 101 times in HepG2 and HT29 cell lines, respectively. The corresponding figures for kunitz4-c were 109 and 286 (Fig. 8a, b). Expression levels of the *P27* (CDKN1B) gene in HepG2 and HT29 cell lines were increased 0.04 and 172 times for kunitz4-a and 6.5 and 188 times for kunitz4-c, respectively (Fig. 8a, b). However, following treatment with kunitz4-a, the expression level of *CDK2* was found to be significantly decreased (*p* = 0.015) in the HT29 cell line and decreased but not significantly in the HepG2 cell line. With kunitz4-c treatments, no significant difference in *CDK2* expression was found in either cell line.

As shown in (Fig. 8d–f) following treatment with kunitz4-a and kunitz4-c, the protein level of *P27* (22 kDa) obviously increased in both HT29 and HepG2 cell lines compared to the controls (untreated cells). Notably, the normal cells are affected by the peptides to a lesser extent.

Likewise, *P53* expression, with an approximate molecular weight of 44 kDa, was markedly increased by kunitz4-a and kunitz4-c in HT29 and HepG2 cell lines, suggesting that both kunitz4-a and kunitz4-c could increase the intracellular expression level of the tumor suppressor *P53* gene in tumor cells.

### Kunitz4 peptides demonstrated a blockade of KV channels

**Figure 9:**
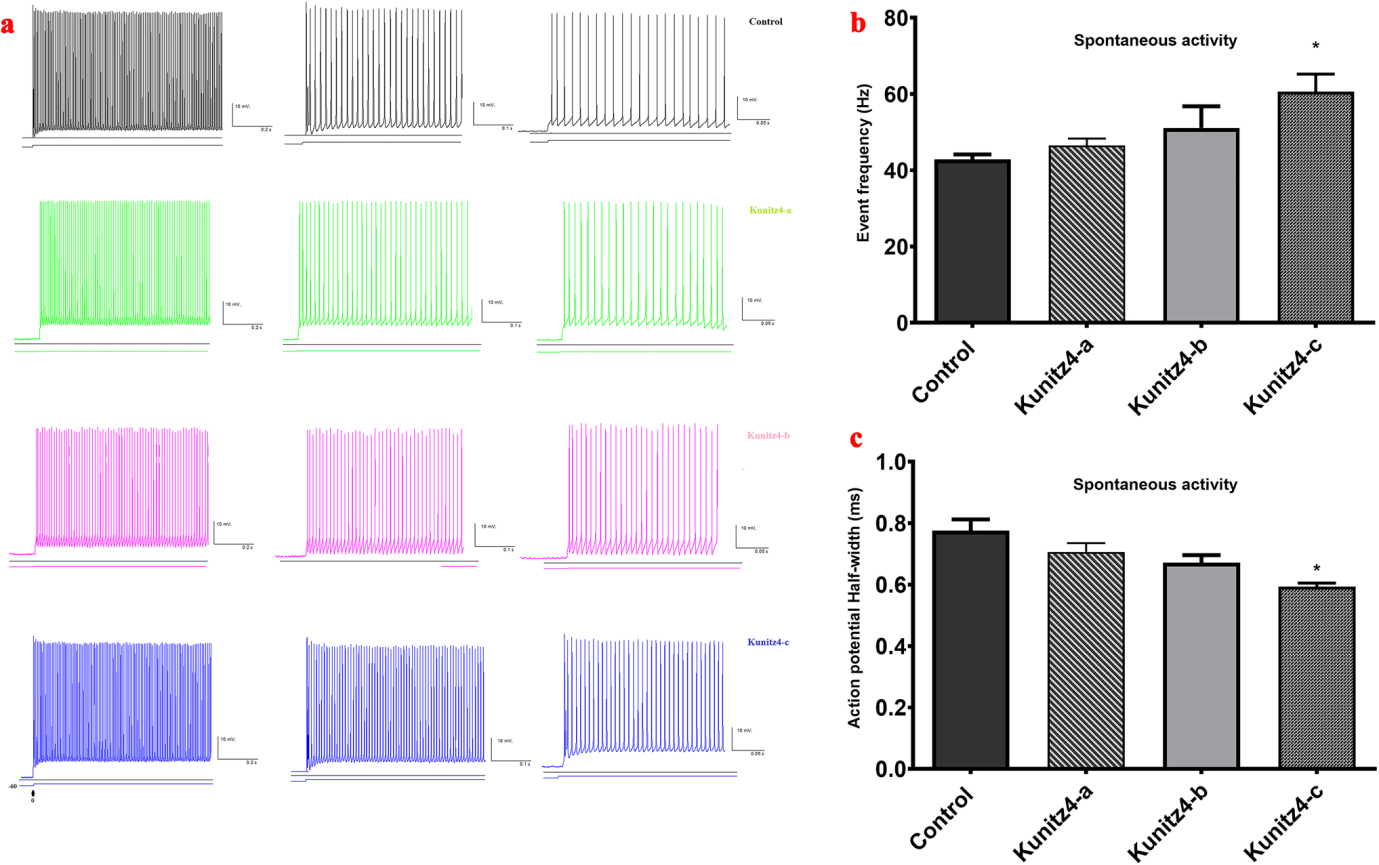
Effects of the small peptides on the spike discharge regularity of cerebellar Purkinje neurons. (**a**) Representative traces of spontaneous spikes recorded from cerebellar Purkinje cells in control and kunitz-treated groups. (**b**, **c**) Purkinje neurons exposed to kunitz-c exhibited an increased firing frequency (**b**) and decreased half-width of action potential (**c**).

Purkinje neurons treated with kunitz4-c demonstrated a significant increase in their spontaneous firing frequency (Fig. 9a), rising from 42.8 ± 1.3 Hz to 60.6 ± 4.5 Hz (F (3, 20) = 3.2, *p* < 0.05; Fig. 9b); however, a significant decrease was observed in their action potential half width, diminishing from 0.77 ± 0.03 ms to 0.59 ± 0.01 ms (F (3, 20) = 8.9, *p* < 0.05; Fig. 9c) when compared to the control rats.

**Figure 10:**
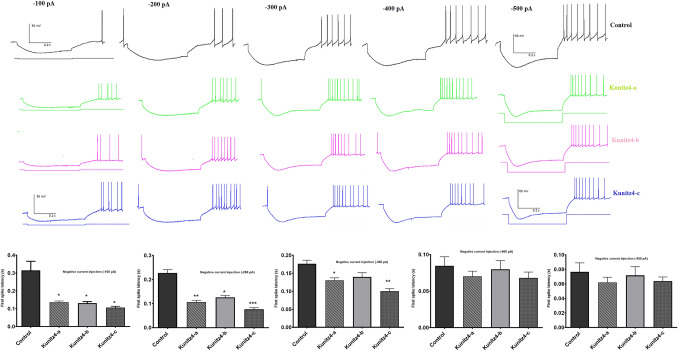
Representative traces of first spike latency and rebound action potentials after injection of negative currents (− 0.5 to − 0.1 nA with 0.1 nA increment, holding voltage =  −  60 mV) plotted as a function of K channels following a hyperpolarizing conditioning pulse (− 100 to – 500 pA). Treatment with the small peptides (kunitz4-a, kunitz4-b, and kunitz4-c) is associated with a significant reduction in the rate of rebound action potentials after the application of hyperpolarizing current pulses.

**Figure 11:**
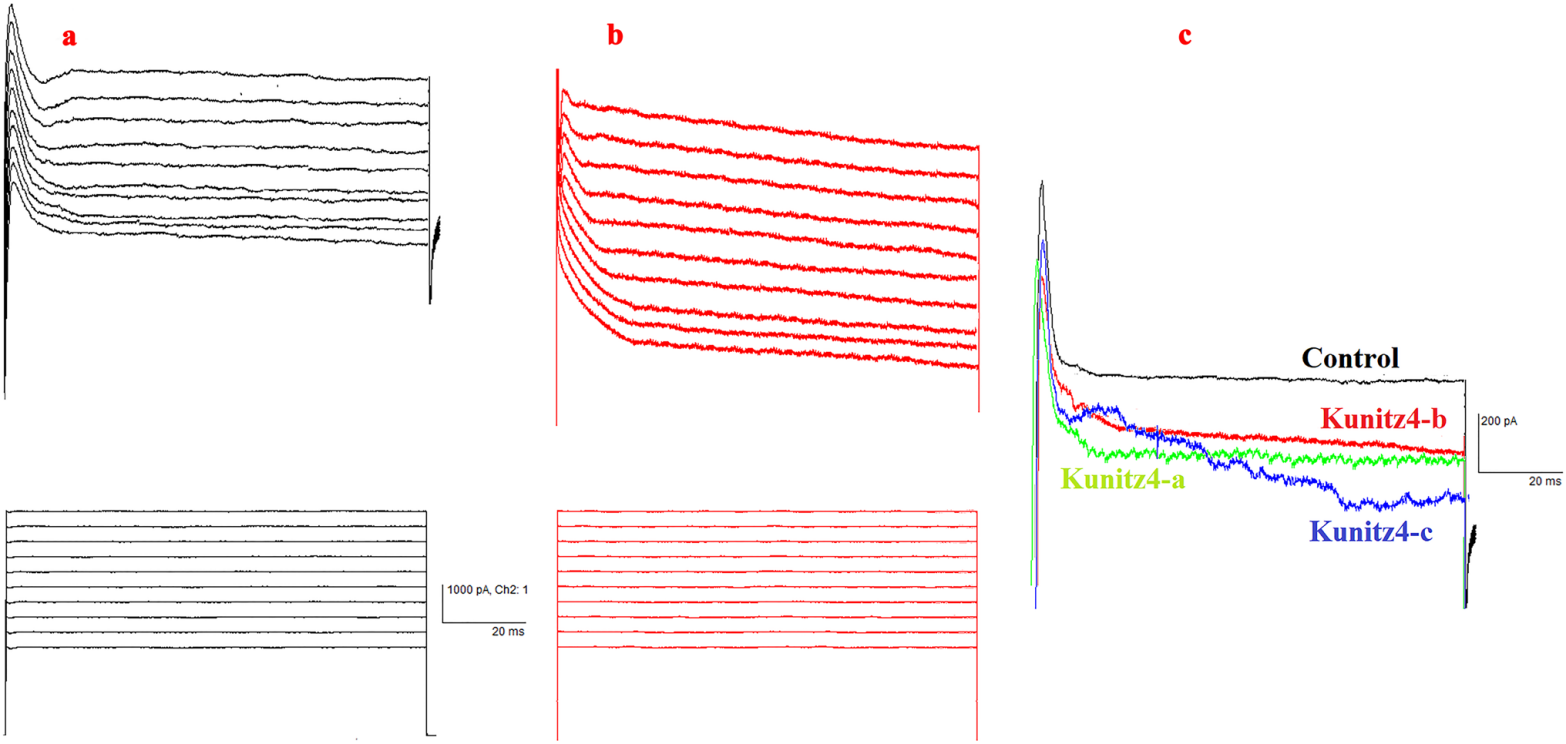
Effects of the small peptides on Kv currents from cerebellum Purkinje neurons. Representative experiments showing that the small peptides (200 nM) block voltage dependent K+ currents elicited by a pulse of − 60 to 30 mV during 100 ms (holding potential Vh = − 60 mV). Effects of the small peptides on K+ currents are activated by increasing voltage pulses. The K+ currents were recorded following stepwise increments of 10 mV of the membrane voltage between − 60 and 30 mV. (**a**) Recordings showing the response of one cell in the control group to elicited pulses, and (**b**) effects of kunitz4-b (200 nM). (**c**) The four traces of four groups are superimposed on an expanded voltage and time scale.

Action potentials were induced in Purkinje neurons using negative current pulses 520 ms in duration and − 0.5 to − 0.1 nA (0.1 nA increments) from *V* holding =  − 60 mV. The first spike latency and rebound action potentials generated by negative current injections were measured as described previously. Purkinje cells from peptide-treated rats in the presence of a hyperpolarizing conditioning pulse (− 0.1 to − 0.3 nA) displayed a significant decrease in the first spike latency (Fig. 10). Exposure to the peptides was also associated with a significant increase in the number of rebound action potentials after the application of hyperpolarizing current pulse − 0.1 nA and a significant increase in the − 0.2 nA only for kunitz-c (Fig. 10). The voltage‑gated K+ current in Purkinje cells was evoked with a step‑up depolarization protocol (Fig. 11a, b). Briefly, the membrane potential was depolarized to + 30 mV (10 mV increments per 10 steps, duration = 100 ms) and subsequently restored to the original potential. Perfusion of 200 μM of each of the peptides (n = 6) produced a slightly significant reduction in peak amplitude (Fig. 11c; command voltage =  + 30: control = 3045 ± 68; kunitz4-b = 2685 ± 51.3, *p* = 0.012; kunitz4-c = 2816 ± 43.1, *p* = 0.032) and the steady-state current (control = 2468 ± 39; kunitz4-a = 2115 ± 34.6, *p* = 0.045; kunitz4-c = 2031 ± 42.1, *p* = 0.04), whereas 200 μM of kunitz4-a (n = 6) produced a non-significant peak current.

## Discussion

In the present study, we demonstrated the anti-tumor activity of Kunitz-type protease inhibitors derived from the tapeworm *Echinococcus granulosus*. Kunitz-type toxins occur naturally in many poisonous animals, such as snakes, sea anemones, and cone snails, and in the saliva of bloodsucking arthropods^22^. The potassium ion channel blocking role of Kunitz-type toxins has been well-documented in collinein-1, the venom of *Crotalus durissus collilineatus*, and Hirudin, derived from the leech, *Hirudo medicinalis* saliva^26,27^. The significance of potassium channels in tumor growth has been clarified in several studies on cancer biology^28^. Current evidence indicates that the upregulation of voltage-dependent potassium channels is connected to cancer hallmarks, and the ectopic expression of Kv10.1 in tumors occurs in the majority of human tumor types^12^.

It has already been shown that the *E. granulosus* Kunitz family demonstrates peptidase inhibitory properties as well as channel blocking effects. Among different members of the family, EgKU-1 and EgKU-4 (Kunitz1 and Kunitz4) induce their effects through voltage-activated potassium channel blockade^24^. In the present study, we characterized the anti-cancer activity of synthetic peptides derived from *E. granulosus* Kunitz4 against two cancer cell lines using computational modeling as well as in vitro cellular methods and biological assays. In addition, protein–protein interactions and Kunitz4 affinity behavior for the EAG1 channel were investigated using molecular simulation. The current findings of modeling showed that synthetic peptides derived from Kunitz4 can induce significant effects equivalent to those of the whole protein.

As therapeutic agents for the incorporation of protein–protein interactions (PPIs), peptides are able to span a large contact surface area, making them suitable for binding to specific cell surface receptors with high specificity and potency; peptide toxins propose a better bioavailability and stability, such as G protein-coupled receptors or ion channels, and consequently, they trigger intracellular targets^29,30^. In designing the peptides, before preparing the complex, we first predicted and validated the proper 3D structure of Kunitz4 as well as the Kunitz4 binding site through multiple ways, such as 3D LigandSite, COACH, and CPORT servers.

Homology modeling of Kunitz4 was performed using 3D conformation of α-dendrotoxin in the venom of green mamba, *Dendroaspis angusticeps* (PDB code: 1dtx). High sequence homology between the toxin and Kunitz4 (e.g., 45% sequence identity between α -DTX and Kunitz4) indicates a similar main-chain fold. Kunitz4 was composed of two α helixes and two anti-parallel β sheets and random coils. Interestingly, sequence analysis of Kunitz4 and 1dtx indicated five cross-linked disulfide bonds between the conserved Cys residues, suggesting that cysteine residues are drastically important to maintaining the tertiary structure. The comparison of α-dendrotoxin and Kunitz4 showed that both molecules share amino acids that can be important for biological activity; for example, Leu27 and Lys47 in Kunitz4 are equivalent to Leu9 and Lys29 in α-dendrotoxin, respectively. These observations signify the potential of Kunitz4 as a cation-channel blocker^24^. Selected docking poses of Kunitz4 amino acids interacting with EAG1 indicated that these amino acids were mostly scattered over the Kunitz4 sequence. Three domains of Kunitz4 are important in interactions with the channel; amino acids within the 21–35 and 42–53 regions of Kunitz4 are involved in the interaction with EAG1, and the third area located between 64 and 76 from Kunitz4 is evolutionarily notable. Along with molecular dynamics simulation, trajectories analysis showed that the predicted structure of Kunitz4 was stable with low fluctuations during the simulation. Moreover, the RMSD plot of the complex of EAG1-Kunitz4 showed convergence after the initial fluctuations. Interestingly, the binding sites of Kunitz4 showed higher fluctuations in the free form compared to the complex with EAG1. Evaluation of the stability of the complex by molecular dynamic simulation showed that the designated peptides interacted effectively in the protein-channel interactions (Fig. 3).

Different K channel-blocking toxins usually share common features essential for interaction with the channel, e.g., functional pairing of the combination of lysine and phenylalanine/tyrosine as hydrophobic residues. This allows the electrostatic interaction of the positive-charged lysine with the negative residues in the pore, causing the physical block of ion conduction, as was shown for Kunitz4 in the present study. Eleven amino acids from Kunitz4 made hydrogen bonds with Kv10.1, and 31 residues were involved in hydrophobic contacts with the channel; most important among them is the conserved lys47 binding to Met478 in the S5–S6 domain. The hydrogen bond triad formed by Arg21, Asn23, and Ile29 with Tyr 344 from the channel made a binding spot that could interrupt the regular octahedral geometry of the open pore.

The crystallography structure of voltage-dependent K+ channel blocking toxins indicated that the effective strategy required a highly conserved residue, Lys47, in the selectivity filter at the extracellular K^+^ binding site^31^. Hotspot residues of EgKU4 computationally predicted with the MM-PBSA approach showed that Leu27 and Lys47 play fundamental roles in PPI (− 17 kJ/mol and – 182 kJ/mol, respectively). Other critical residues constructing three peptides include Arg21, Lys31, Arg 35, Arg 52, Asn61, Arg 64, Lys66, Lys68, Arg 69, Lys72, and Arg 73. Considering the data, the three regions of Kunitz4 were selected for peptide design against the extracellular S5-S6 domain using hotspot residues prediction with MM-PBSA in GROMACS. As is the case in charybdotoxin isolated from *Leiurus quinquestriatus*, the scorpion toxin that fits into the pore of the channel in a lock and key posture^31^, peptides block EAG1 in the same manner (Figure S3).

The first natural toxin inhibiting Kv10.1 (EAG1) was reported from scorpion toxin κ-hefutoxin 1, isolated from *Heterometrus fulvipes*^32^*. *The MIC50 value of the toxin against *Candida albicans*, *Saccharomyces cerevisiae,* and *Fusarium oxysporum* was found to be in the range of 18.8–37.7 µM. APETx4, a peptide derived from the sea anemone *Anthopleura elegantissima*, as a gating modifier, has been shown to induce inhibitory effects on EAG1 through binding to the S3‐S4 region. Furthermore, it has displayed concentration-dependent proapoptotic and cytotoxic effects in LNCAP, SH-SY5Y, and MDA-MB-435S cancer cell lines^33^. Collinein-1, a snake venom thrombin-like enzymes (SVTLEs) reduced the viability of the human breast cancer cell line MCF7 but did not affect either the HepG2 cell line or MCF10A, the non-tumorigenic epithelial breast cell line. Arg79 residue is essential for the Kv inhibitory motif and interacts directly with the EAG1 channel selectivity filter^27^. In a xenograft model of liver cancer, Procyanidin B1, a natural composition from the grape seed, was identified as a specific inhibitor of Kv10.1; it inhibited tumor growth in vivo and suppressed proliferation and migration of hepatoma cells^34^. Herein, we evaluated the anti-proliferative, cytotoxic, and proapoptotic effects of Kunitz4-derived peptides on cancerous and normal cell lines. The peptides were shown to be able to induce a dose-dependent effect on both cancer cell lines; however, comparing to HepG2 and Ht29, very low anti-proliferative and cytotoxic effects were found on Hek293, the normal control cell line. It should be noted that the cytotoxic effect was remarkably higher on colorectal adenocarcinoma cell line HT29 than HepG2, the hepatocarcinoma cells, with lower IC50s. An increased apoptosis rate was also observed after treatment of the cells with certain concentrations of the peptides. As shown in Fig. 7, the cancer cells displayed typical apoptotic features; however, minimal changes were seen in noncancerous Hek293 cells.

Much evidence has shown that potassium channel activity is required for G1 progression of the cell cycle in different cell backgrounds, suggesting that potassium channel activity is necessary for early-stage cell proliferation through the G1 phase and facilitation of G2/M progression during the cell cycle. Moreover in cancer cells, the activity of EAG1 is high in the G1 phase and decreases when the cells enter the S phase^35^. The current findings showed a significant accumulation of cancer cells in the G0/G1 phase as well as decreases in cells in the G2/M and S phases in comparison to the control cells (Fig. 7). The results suggest that cell proliferation is suppressed by the peptides through cell cycle arrest at the G0/G1 phase, providing a chance to repair and discontinue proliferation of damaged cells.

Notably, high expression of *P53* is involved in the regulation of cell cycle arrest. Potassium channels are involved in apoptosis by regulating cell volume; when the regulation fails, the outflow of K+ ions become too intense, water leaves the cells, and that results in a decrease in cell volume and subsequent cell death. *P53* is a master regulator of DNA replication and cell death in cancer cells and controls the three checkpoints G1, S, and G2/M by regulating CDKs and cyclins as positive and *P16*, *P27* and *P21* as negative regulators of the cell cycle. In the present study, by comparing responses of the two cell lines with the corresponding controls, the synthetic peptides showed a significant impact on the cell cycle following upregulation of *P53.* The same trends were found in cell cycle arrest caused by the peptides. These results demonstrated that kunitz4-a and kunitz4-c could probably inhibit the downstream signaling through cell cycle arrest involving the up-regulation of *P53* and *P27*. Our findings are in line with the fact that the tumor suppressor *P53* gene induced cell cycle arrest at the G0/G1 phase. Therefore, p53-triggered inhibition of cell proliferation could be attributed to cell apoptosis and induction of cell cycle arrest^36^. The expression of *P27* in both HepG2 and HT29 cells was remarkable in this study, and the distribution of HepG2 cells in the cell cycle showed that the peptides significantly increased the cells in the G0 and G1 phases, while cells in the S and G2-M phases were decreased. *P27,* a main cell cycle regulator, accelerates cell cycle development in HT29 and HepG2 cells; therefore, a 2- to 3-fold increase in *P27* expression is adequate for obstructing the G1–S-phase and inhibiting cyclin-CDKs^37^. TEA, 4-AP, and glibenclamide blocking the Kv and K_ATP_ channels in the U87 MG glioma cell line could inhibit tumor growth by arresting G0/G1 transition^38^. The current study demonstrated that overexpressed *P53* and *P27* downregulated Ki-67 and induced apoptosis. On the other hand, an inverse correlation was observed between *P53* expression and proliferative activity evaluated by Ki-67 protein using ICC. The Ki-67 protein, with its relatively short half-life is present during all active phases of the cell cycle (G1, S, G2, and M). During anaphase and telophase, a firm decrease in Ki-67 levels occurs. Expression of the Ki-67 protein, as a marker of tumor aggressiveness, is associated with the proliferative activity of malignant tumor cell populations, as it has been shown in a number of studies on cancers of the cervix, lung, prostate, breast, soft tissues and the central nervous system^39,40^. The present study demonstrated that kunitz4-a and b decreased *CDK4* mRNA in HepG2 cells, while only kunitz4-c caused an increase in the expression of *P27;* the other peptides had no significant effect on *P27* in HepG2 cells (Fig. 8). In HT29, however, this condition was different, and all the peptides could produce significant amounts of *P27* and reduce *CDK2* expression with no considerable effect on *CDK4*. These findings indicate that the peptides inhibit cell proliferation by inducing G1 arrest through accumulative effects of *P27* and *P21*, two well-known CDK inhibitors regulating cell proliferation41. Huang reported that treating the U251 glioma cell line with K_ATP_ channel blockers resulted in cell cycle blockage in the G0/G1 phase^10^. In another study, G2/M cell cycle blockage and apoptotic cell death were documented following Ca2+ -activated Kv channel KCa3.1 blocking in GL261 glioma cells treated with temozolomide^42^. The present study provides evidence of change in gene expression at the RNA level of *P53* and *P27* caused by some peptides in tumor cells, however, this difference does not translate to the protein level which is expectable because of the extensive regulation of these proteins, especially *P53*, at the posttranslational level. It should also be noted that, other factors determining the specificity of the Kunitz4 peptides towards cancer cells need to be further studied.

Cancer cells have more positive membrane potentials than normal cells. It has been demonstrated that cells are depolarized in the early G1 phase and hyperpolarized during the progress from the G1 into the S phase^41^. As shown in Figs. 7, 9, and 11, inhibition of K+ channels or cell blockade in the G1 phase is associated with membrane depolarization. Likewise, membrane depolarization initiated by an increase in the concentration of extracellular K+ simulates the effects of potassium channel blockers. Alterations in the firing characteristics of Purkinje cells are reflected in the functional properties of ion channels, which provide basic regulation of nerve excitability. The current results showed that treatment with Kunitz4 peptides increased the Purkinje neuronal intrinsic excitability, which is thought to be a function of inhibition potassium channels, especially by kunitz4-c. Purkinje neurons exposed to peptides exhibited significantly shorter duration of action potential and increased action potential frequency.

Treatment with peptides also induced a significant decrease in latency to the first spike that was associated with a significant increase in the rebound spike firing at the offset of hyperpolarization. This is in accordance with findings of previous studies which have revealed that the blockade of KV channels affects not only the first spike latency, but also the after hyperpolarization and frequency of spikes^43^. The shortening of the first spike latency could be attributed to the inhibition of the fast transient (A-type) channel, which is thought to play a key role in the timing of rebound action potentials^44^. The three peptides synthesized based on *E. granulosus* Kunitz4 showed anti-cancer activities against two human cancer cell lines, namely HepG2 and HT29. Cellular and molecular findings based on cell viability, cell cycle and apoptosis analyses, immunocytochemistry, major cancer cell gene involvement in cell progression, and whole-cell patch clamp recordings showed that the peptides have highly selective inhibitory effects on A type channels and are capable of suppressing tumor cell growth by inducing cell cycle arrest and apoptotic cell death. However specific actions of the peptides through the inhibition of K+ channels (specifically EAG1 channel) should be substantiated by the correlation of EAG1 protein level and activity with the effect of the peptides in several cell lines. The peptides characterized and evaluated in the present study have a promising potential for further in-depth cellular and molecular investigations in both in vitro and in vivo settings.

## Methods

### Sequence analysis of Kunitz4 protein and its homologs

The Kunitz4 protein sequence of *Echinococcus granulosus* G1 genotype was extracted from NCBI (APY21165.1), and the sequences of the dendrotoxin I from *Dendroaspis polylepis polylepis* (black mamba) (NCBI:P00979), α-dendrotoxin from the green mamba venom (NCBI:P00980), *Dendroaspis angusticeps* (eastern green mamba), (NCBI:P00980), homolog K from *D. polylepis polylepis* as dendrotoxin k (NCBI:P00981), and the Kunitz domain of tissue factor pathway inhibitor (NCBI:P10646) were used to compare Kunitz4 and sequence alignment analysis of the toxins as well as for the homology modeling of *Echinococcus granulosus* Kunitz4.

### Kunitz4 structure determination

Based on the aforementioned templates, the three-dimensional protein structure of Kunitz4 was predicted by a model constructed from its primary amino acid sequence (NCBI: APY21165.1) using Modeller 9.22. Structure validation was performed using MolProbity^45^, ProSA^46^, ERRAT, and Vadar web servers^47^. The reliability of the homology model was assessed by calculating the Z-score and Ramachandran plot. The hydrophobicity plot was obtained in Discovery Studio 2.5 according to amino acid residue in the favored regions^48^.

### Active site prediction

3D LigandSite, CPORT (https://milou.science.uu.nl/services/CPORT), and COACH (https://zhanglab.ccmb.med.umich.edu/COACH) were the programs utilized for predicting protein–protein interface residues. Among the noted, six interface prediction methods were combined into a consensus predictor by CPORT, and the predictions were used as active and passive residues of Kunitz4 protein in molecular docking.

### Molecular docking

Molecular docking was performed by using HADDOCK 2.4 and Cluspro 2.0 protein–protein docking programs. Docking was simulated to the extracellular domains of the cryoEM of Kv10.1 (PDB ID: 5K7L) as a template to construct a continuous Kv10.1 channel structure. M-ZDOCK was utilized to predict the complementarity tetramer of Kv10.1 based on the structure of an unbound or partially bound monomer structure. Finally, each pose was weighted based on energy scores as described in detail by Chen and Weng. As a flexible docking approach, HADDOCK encodes information from predicted protein interfaces for the modeling of complexes^49^. We used Cluspro 2.0 in the rigid body docking step as another docking program which uses PIPER, an FFT-based docking algorithm. The Kunitz4 protein and EAG1 active site residues were defined for docking (Supplementary Dataset File 1). To this end, the highest scores were analyzed to identify the best channel-toxin-like conformation. Using Discovery Studio 2.5^48^ and Ligplot+^50^, all top-scoring conformations of every protein docking server were analyzed in terms of the number of hydrophobic and H-bonds. Finally, the top-ranking complex of EAG1-Kunitz4 models was subjected to 50,000 ps MD simulations and alanine mutation based on molecular mechanics/Poisson–Boltzmann surface area (MM-PBSA). To check the molecular interaction of EAG1-peptides, molecular docking was carried out using ClusPro and HADDOCK. EAG1-peptide complexes were subsequently analyzed by Ligplot+ and Chimera. The complexes with the lowest interaction energy and the highest interaction bonds were selected for channel inhibition. Using the PRODIGY server, the binding affinity (ΔG) and dissociation constant (Kd) were calculated for selected peptide complexes.

### Molecular dynamics simulations

The stability and conformation of the docked complexes and 3D structure of Kunitz4 were determined using the program package GROMACS 5.1.4^51^ along with gromos54a7 ff force field. The system was solvated with TIP3P water and simulated in an octahedron box with periodic boundary conditions (11 Å padding in each direction). Then the net charge system was neutralized by adding sodium and chloride ions to the protein. Minimization was performed with 5000 steps by the steepest integrator, and the maximum force was less than 1000 kJ/mol/nm. Subsequently, the system was equilibrated for 1 ns in the NVT and a constant pressure (NPT) of 1.0 bar. The LINCS algorithm was applied to all bonds involving hydrogen atoms during the heat and constant pressure steps. Force constants of 1000 kJ/mol/nm^2^ were employed during the equilibration phase. Afterwards, the isothermal-isobaric MD simulation produced a 50,000-ps trajectory with a non-bonded cutoff distance of 14 Å and a leapfrog integrator with a 2.0 fs time step. All bonds involving hydrogen atoms were constrained by the LINCS algorithm during the heating and the constant pressure steps. Using the Particle Mesh Ewald method, the electrostatic interaction energies were calculated, and the energies and coordinates of each atom were stored every 2 ps for subsequent analyses. Regarding the system behavior, RMSD, RMSF, Rg, SASA, and number of hydrogen bonds in the Kunitz4 and EAG1 interface were extracted from trajectories. The data was visualized using GraphPad Prism version 9.0.0.

### Alanine mutation and binding free energy calculations

To calculate free energy of binding (ΔG) and electrostatic energy, VdW energy, polar solvation energy, and surface accessible surface area, we used the Molecular Mechanics Poisson-Boltzmann Surface Area (G_MMPBSA) process executed in GROMACS 5.1.4^52^. A total of 38,000 snapshots of structures at intervals of 10 ps were extracted from 50,000 frames of the MD runs and used to calculate the system enthalpy using MmPBSADecomp.py script provided along with the g_mmpbsa tool.

### Peptide design

Three distinct sites from Kunitz4 that interact with EAG1 were selected for peptide design. A peptide was constructed based on all possible amino acid substitutions. Key residues were recognized in Kunitz4-EAG1 interactions according to molecular docking studies, literature data mining, and multiple sequence alignment of the target to find conserved areas. Most processes involving peptide interactions were constructed using molecular dynamics-based methods as well as free energy calculations of snapshots equally spaced along a single dynamical trajectory^53^. Using the calculations, the experimental ΔΔG of binding with a mean error of ± 1 kcal/mol was predicted for the alanine mutations of hydrophobic and polar/charged residues with no buried salt bridges.

### Evaluation of physicochemical properties of the peptides

The physiological/biochemical properties of the peptides, including molecular weight, isoelectric point, aliphatic index, water solubility, net charge at pH 7, grand average of hydropathicity (GRAVY), aggregation hot spots, and instability index, were calculated using ProtParam and PepCalc^54^. A negative GRAVY value indicates hydrophilicity, while a positive value indicates the hydrophobicity level of a protein. The best peptides with completely matched properties for in vitro experiments were selected based on the binding affinity energy, predicted dissociation constant (Kd), and stability (Table 2).

**Table 2:** Physicochemical properties, instability index and binding energy of the Kunitz4 derived peptides.

Peptide name	Binding energy (kcal/mol)	GRAVY^a^	Net charge at PH 7	Water solubility	Instability index^b^	Isoelectric point	HPLC purity (%)	Salt form
Kunitz4-a	− 770.7	− 0.900	2	Good	117.23	11.21	95.28	Acetate
Kunitz4-b	− 1673.8	− 1.283	0.9	Good	35.83	8.77	96.31	Acetate
Kunitz4-c	− 1488.5	− 2.046	5.1	Good	80.85	11.9	95.28	Acetate

^a^Positive GRAVY values indicate hydrophobic; negative values mean hydrophilic.

^b^Instability index value < 40 indicates that the peptide is stable.

### Synthesis of peptides

Three segments of a whole Kunitz4 inhibitor protein of E. granulosus, including a 15 mer kunitz4-a, 12 mer kunitz4-b and 13 mer kunitz4-c, were selected for in vitro study. To improve chemical stability and solubility, a mini-PEG-2 was added at the N terminus of the kunitz4-a peptide. All peptides were synthesized at a final purity > 95% HPLC grade and < 1% TFA salt by Biomatik Co. Ltd. (Canada).

### Cell culture

Three cell lines, i.e. HT29, a human colorectal adenocarcinoma cell line with epithelial morphology; HepG2, a human liver cancer cell line; and Hek293, a human embryonic kidney 293 as the normal control cell line, were obtained from Stem Cell Technology (BonBioTech, Iran). The HT29 and HepG2 cancer cell lines as well as the Hek293 cells were maintained in Dulbecco’s modified Eagle medium supplemented with 10% (v/v) fetal bovine serum and 1% (v/v) penicillin/streptomycin. All media and bovine serum were purchased from Cegrogen-Biotech (Stadtallendorf, Germany). All cells were seeded onto 25-cm^2^ culture flasks (T25 Flask, SPL Life Sciences) and incubated under a humidified atmosphere of 95% air/5% CO2 at 37 °C.

### Cell proliferation assay

Approximately 5000 cells/well of HT29, HepG2, and normal Hek293 cell lines were plated into 96-well microplates and incubated in various concentrations (0 μM up to 800 μM) for 12, 24, and 48 h, each treatment was prepared in triplicate. Peptide stock solutions at a concentration of 1 mM were prepared and further diluted in the medium to achieve final concentrations of 100–800 µM. Different peptide concentrations in the growth media were added to the cells, and the no-peptide negative control was treated with an equal volume of media. Following peptide treatments, MTT solution (10 µL of 5 mg/mL in 0.01 M sterile PBS, pH 7.4) was added to each well, and the plate was incubated for 3 h at 37 °C in 5% CO_2_. After removing the medium, 100 µL/well DMSO was added and the plates were shaken for 10 min to dissolve the formazan. Using a microplate reader (BioTek-ELX800, USA) ODs were measured at 450 nm.

### Apoptosis analysis

An Annexin V/PI Apoptosis detection kit (cat. no. AnxF100PI, MabTag GmbH) was used to evaluate apoptosis after peptide treatments. At a density of 3 × 105 cells/mL, a volume of 2.5 mL cell suspension/well was seeded onto a 6-well plate. Following 24-h cultures of HT29 cells in 100 µM, and HepG2 and Hek293 cells in 250 µM of each peptide (kunitz4-a, kunitz4-b, and kunitz4-c), the cells were treated as described in the previous section. The negative controls were treated with equal volumes of PBS. After incubation, cells were harvested and washed twice in cold PBS, pH 7.4, resuspended in Annexin-binding buffer and incubated with 5 μl FITC-Annexin V and 5 μl PI. Samples were vortexed and incubated in the dark at 25 °C for 15 min. The stained cells were analyzed using a Sysmex Cyflow Space flow cytometer, and following excitation at 488 nm, fluorescence was measured at 495 nm and 519 nm.

### Cell cycle analysis

Following several repetitions, the minimum concentration values were chosen to investigate the effects. After 24 h incubation of HT29 cells in 100 µM and HepG2 and Hek293 cells in 250 µM of each peptide, cells were harvested by trypsin and washed twice with PBS before being fixed in 1 mL cold ethanol (70% v/v) at − 20 °C overnight. After removing the fixative solution and another round of PBS washing, the fixed cells were re-suspended in PI staining solution and placed at 4 °C in the dark for at least 2 h. FlowJo software was used to analyze cell proportions in each phase. All experiments were performed in triplicate, and the results were presented as percentages of cells in each phase.

### Expression of *P53*, *P27*, *P16*, *CDK2*, and *CDK4* genes

After 24 h of treatment of HepG2 and Hek293, with 250 µM and HT29 cells with 100 µM of each peptide, total RNA was isolated using the TRIzol protocol. An equal amount of total RNA was used to synthesize cDNA using an Easy cDNA Synthesis Kit (Pars Tous, Mashhad) as instructed by the manufacturer. The expression of five major cancer cell biomarkers, namely *P53*, *P27*, *P16*, *CDK2*, and *CDK4* genes, were determined by qRT-PCR. Two-step PCR conditions were used with the following thermal profile: 95 ℃ for 3 min, 40 cycles of 95 ℃ for 6 s, and 60 ℃ for 40 s. For each cell line, two internal standard genes were used as follows: *GAPDH* and *PGK1* for HT29, *GAPDH* and *TFG* for HepG2, and *HPRT* and *GAPDH* for HEK293^55^(Supplementary Table 2). Data was statistically analyzed using the 2−∆∆Ct method^56^.

### Immunocytochemistry staining

Immunocytochemical analysis was performed on the detached cells. Smears were prepared on adhesive, positively-charged slides after 24 h of incubation of HepG2 and HT29 cells with 250 µM and 100 µM, respectively, of kunitz4-a and kunitz4-c. The slides were washed three times with PBS after 1 ml pure ice-cold acetone/methanol (1:1) was added to the slides at − 20 °C. Immunostaining was performed with 1:200 diluted mouse anti-Ki-67 monoclonal antibody (Zytomed, mouse anti-Ki-67, MSK018), and the slides were incubated for 30 min at room temperature. Subsequently, secondary HRP antibodies were used (Zytomed, Germany) with incubation times of 30 min and counterstained with hematoxylin (Dako, Waldenbronn, Germany) for a few seconds. The cells were analyzed and photographed at × 400 magnification using a digital microscope camera coupled with a computer (Olympus BX53; Olympus, Tokyo, Japan). To analyze immunoreactivity, three slides from each experimental group and 20 fields of each slide were examined to acquire the intensity of reactions.

### Western blotting

Protein expressions of *P53* and *P27* were evaluated using Western blotting as described by Liu and Bodmer^57^. The proteins were electrophoresed (100 V, 2–3 h) in 12% sodium dodecyl sulfate polyacrylamide gels. The separated proteins were electro-transferred to nitrocellulose membranes using the Trans-BlotVR Turbo Transfer system (Bio-Rad-20 V, 60 min). Afterwards, the membranes were blocked (1 h) using 3% bovine serum albumin (BSA) in Tris-buffered saline, pH 7.4, and washed several times using TBS buffer. The membranes were subsequently probed with 1:1000 primary antibody *P53* (GTX50438) and *P27* (GTX27961) for 2 h in TBS buffer and washed several times (TTBS, 10 min each). Finally, HRP-conjugated anti-rabbit IgG antibody (GTX213110-01) was used to detect primary antibodies. To develop blots, the membranes were exposed to enhanced chemiluminescence (ECL). GAPDH (GTX100118) was used to normalize protein expression.

### Whole-cell patch clamp recording

The effects of the peptides on the electrophysiological properties of the Purkinje cerebellum neurons were evaluated by whole-cell patch clamp recordings. As described elsewhere^58^, rat brains were quickly removed and placed in ice-cold artificial cerebrospinal fluid ACSF containing NaCl (124.0 mM), NaHCO3 (25 mM), d-glucose (10 mM), KCl (4.4 mM), MgCl2 (2 mM), KH2PO4 (1.25 mM), and CaCl2 (2 mM), with the osmolarity adjusted to 300 mOsm and bubbled with 95% O_2_-5% CO_2_ (pH 7.3–7.4). The cerebellar vermis was dissected and parasagittal slices of 250 μm thicknesses were cut by vibroslicer (Campden Instrument, NVSLM1, Sarasota, FL, USA). The slices were continuously superfused at 2 ml/min with ACSF. Using multiclamp 700B amplifiers (Axon Instruments, Foster City, CA), intracellular recordings in current and voltage clamp modes from Purkinje neurons were made, and signals were digitized by Digidata 1320 A/D converter (Axon Instruments, Foster City, CA). The patch pipettes had a resistance of 4–8 MΩ when filled with internal solution containing KMeSO4 (135 mM), KCl (10 mM), HEPES (10 mM), MgCl2 (1 mM), Na2ATP (2 mM), and Na2GTP (0.4 mM).

### Statistical analysis

The data analysis and plots were performed in GraphPad Prism version 9.0.0. The error bars on the figures correspond to the SEM. Statistical analysis was performed using the Student’s t test or one-way ANOVA and two-way ANOVA method with Tukey’s post-test, comparing all treatments to the negative controls and considering values of P values < 0.05 were considered significant. n represents the number of recordings.

## Supplementary Information

Supplementary Information 1.

Supplementary Information 2.

Supplementary Figure 1.

Supplementary Information 4.

## Data Availability

The used and/or analyzed datasets during the current study are available from the corresponding author on reasonable request.
