# Supplementary figures and images for: Design of ion channel blocking, toxin-like Kunitz inhibitor peptides from the tapeworm, Echinococcus granulosus, with potential anti-cancer activity
Source: Sci Rep. 2023 Jul 15;13:11465. doi: 10.1038/s41598-023-38159-w (PMC10349847; doi:10.1038/s41598-023-38159-w)

## Slide 1
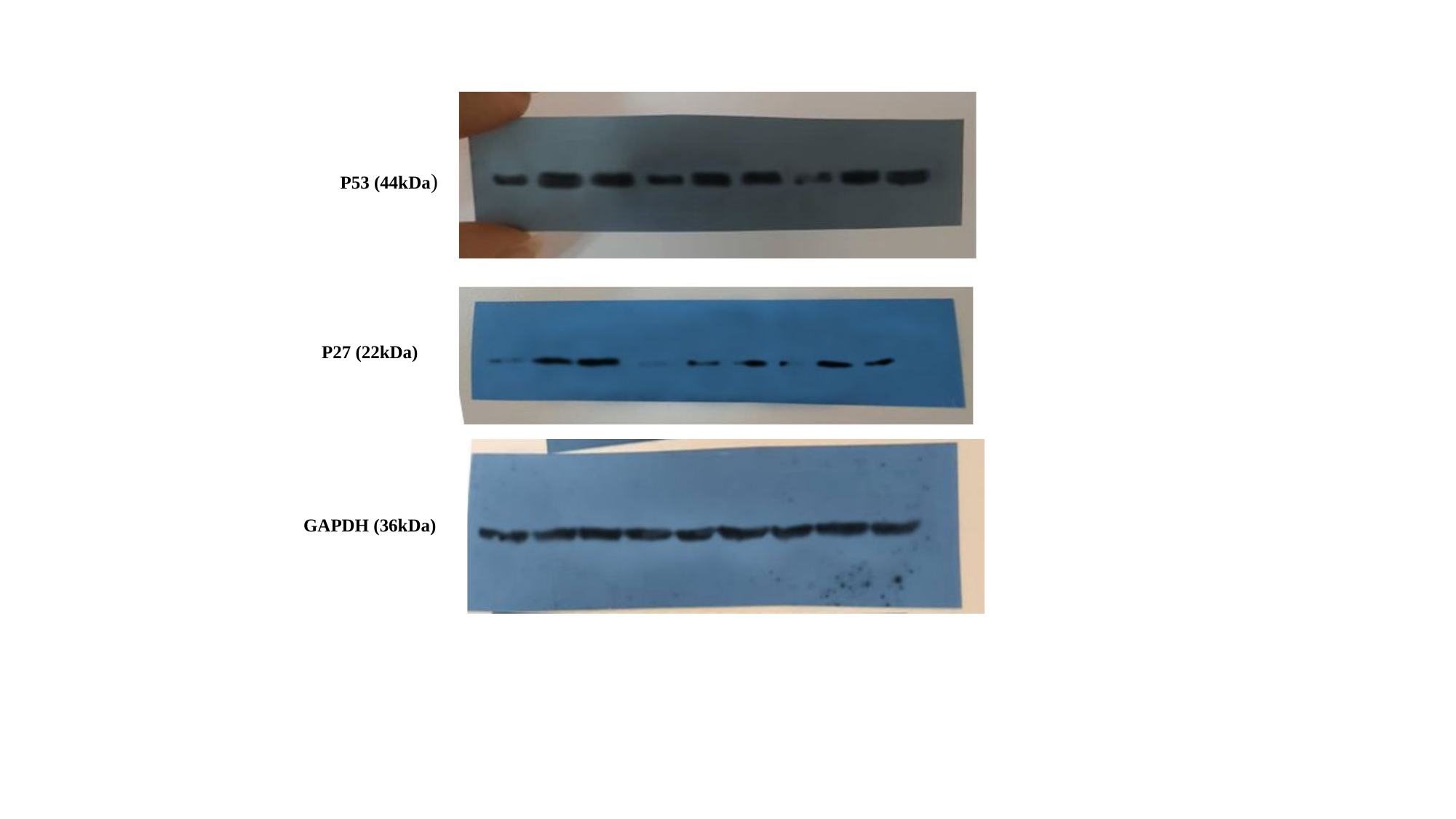

P53 (44kDa)
P27 (22kDa)
GAPDH (36kDa)

Supplement: Supplementary file 3 — Supplementary Figure 1. [file 41598_2023_38159_MOESM3_ESM.pptx]
