# Supplementary material for: Design of ion channel blocking, toxin-like Kunitz inhibitor peptides from the tapeworm, Echinococcus granulosus, with potential anti-cancer activity
Source: Sci Rep. 2023 Jul 15;13:11465. doi: 10.1038/s41598-023-38159-w (PMC10349847; doi:10.1038/s41598-023-38159-w)
Supplement: Supplementary file 4 — Supplementary Information 4. [file 41598_2023_38159_MOESM4_ESM.pdf]

Supplementary Table 1. Structure-validation of stereo chemical properties of the predicted model of Kunitz4 by homology modeling.

MolProbity clash score*	ERRAT(%)**	Ramachandran plot(%)				Free energy of folding(kcal.mol^{-1})	Confidence and P-value	ModFOLD Global quality score
		Ramachandran Favored	Additionally allowed	Generously allowed	Disallowed			
75.78	80	94.74	3	2	0.0	-52.94	1.394E-4***	0.5500****

*The 'clashscore' is the number of serious steric overlaps (>0.4 Å) per 1000 atoms.
**Expressed as the percentage of the protein for which the calculated error value falls below the 95% rejection limit. Good high resolution structures generally produce values around 95%
***p < 0.001, Less than a 1/1000 chance that the model is incorrect.
****Scores greater than 0.4 generally indicate more complete and confident models, which are highly similar to the native structure.

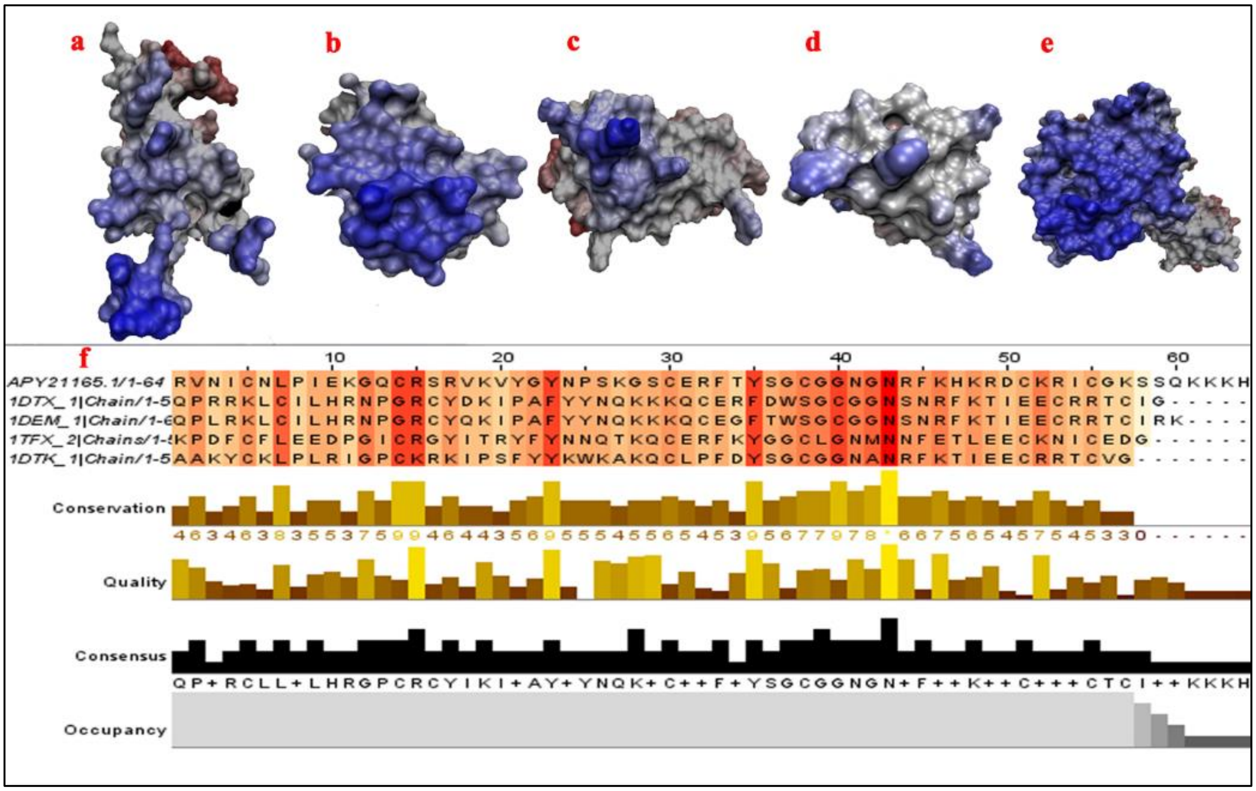

Figure S1. Structure analyses of *E. granulosus* Kunitz-4 alignment and its homologues. (a-e) Molecular surface electrostatic representations of the same proteins in the same orientation, highlighting global differences in charge distribution; scale represents charge from positive blue to negative red. (f) Comparison of the full-length amino acid sequences of Kunitz-4, 1dtx,1dem, 1dtk, 1tfx. The alignment was constructed using Clustal W2.

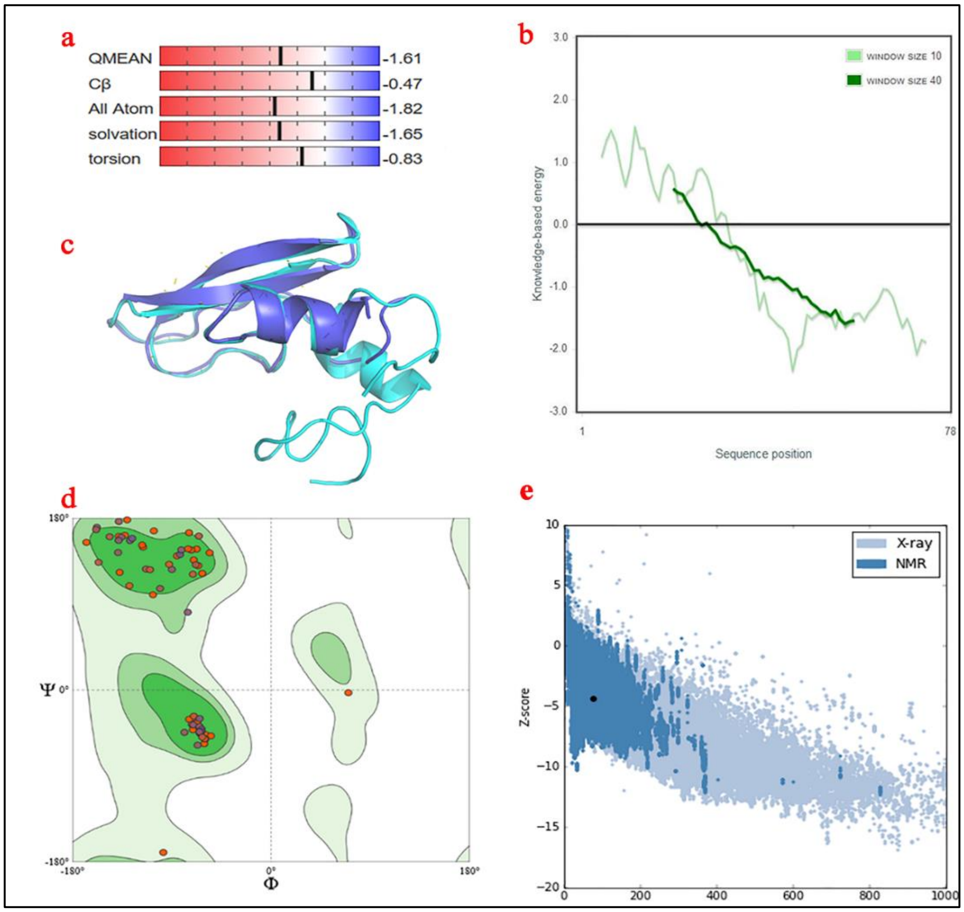

Figure S2. Validation of Kunitz4 model by several methods. (a) Quality Estimate of Kunitz4 with SWISS-MODEL, the QMEAN is a composite estimator based on different geometrical properties and provides both global (i.e. for the entire structure) and local (i.e. per residue) absolute quality estimates on the basis of one single model. (b) Local model quality by plotting energies as a function of amino acid sequence position. Generally, positive values correspond to problematic parts of the input structure. (c) 3D structure alignment of Kunitz4 and 1dtx. Kunitz4 was colored in cyan and 1dtx in blue. (d) Ramachandran plot. The most favored, additionally allowed, generously allowed and disallowed regions are shown in green, pale green, gray and white colors, respectively. (e) ProSA Z-score plot of modeled 3D structure of Kunitz4. The position of this model among experimentally solved protein structures is shown in black circle.

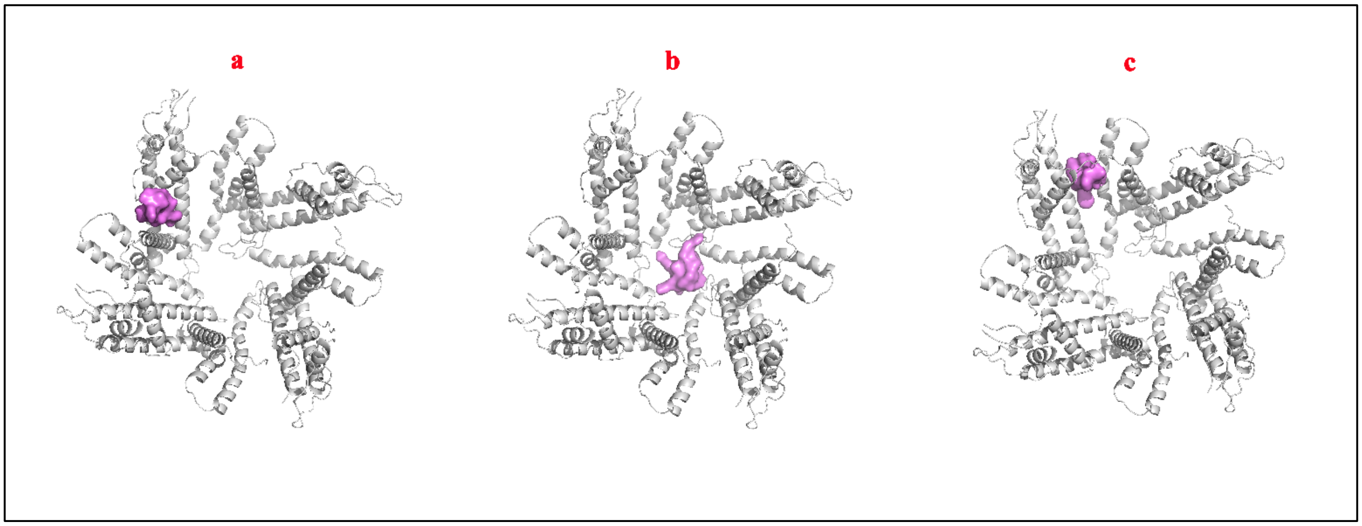

Figure S3. Cartoon and surface representations of the EAG1 and the three peptides complex, kunitz4-a (a); kunitz4-b (b) and kunitz4-c (c).

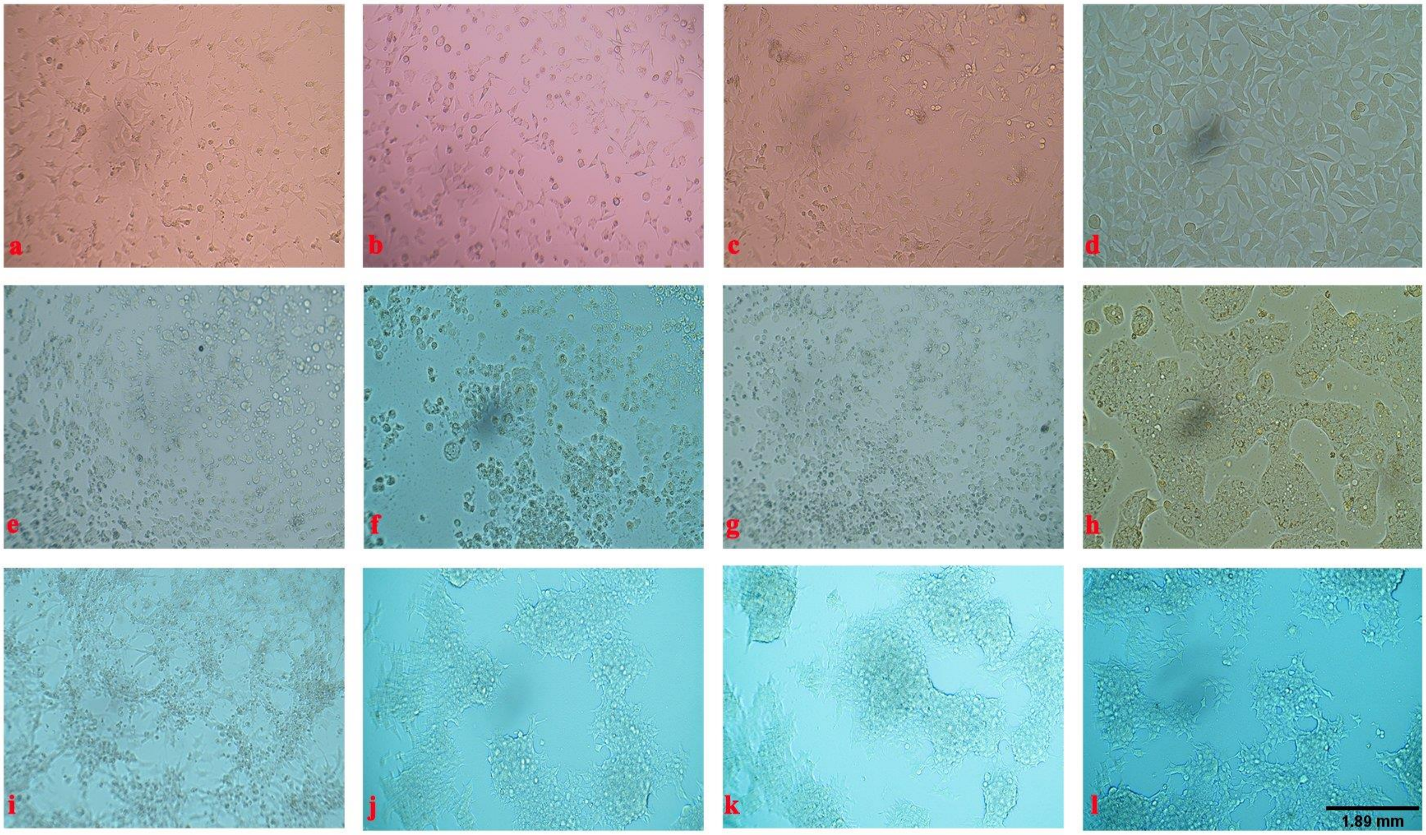

Figure S4. Effect of the synthetic peptides on the cellular morphology of HepG2 (a, kunitz4-a; b, kunitz4-b; c, kunitz4-c; d, untreated), HT29 (e, kunitz4-a; f, kunitz4-b; g, kunitz4-c; h, untreated) and Hek 293 (i, kunitz4-a; j, kunitz4-b; k, kunitz4-c; l, untreated).

Supplementary Table 2: Primer sequences (Eurofins genomics, Germany) used for qRT-PCR

Gene name	Forward (5' - 3')	Reverse (5' - 3')
KCNH1	GTTCGGCGGTCCAATGATACT	GCCAGACAGCTTGCAAAATCC
CDK2	ACAAAGCCAGAAACAAGTTGACG	CCCTCAGTCTCAGTGTCCAGG
CDK4	GTCTATGGTCGGGCCCTCT	AGGGAGACCCTCACGCCA
TP53	ACAACGTTCTGTCCCCCTTG	CTGGCATTCTGGGAGCTTCA
GAPDH	GTGACTAACCCTGCGCTCC	CGCCCAATACGACCAAATCAGA
CDKN1A	GGTGTTTCTGCGGCAGGC	GGCCATTAGCGCATCACA
CDKN2A	TAGAAGCAGGCATGCGTAGG	ATCGGGGATGTAATGCCAGG
HPRT1	GCCCTGGCGTCGTGATTAG	TCTCGAGCAAGACGTTCAGTC
PGK1	GAGATGATTATTGGTGGTGGAA	AGTCAACAGGCAAGGTAATC
TFG	CTTGGGGAGGATATTCGGCG	GGCGCTTGTTATAAGATCTCCATC
